# Clinical prediction model for transition to psychosis in individuals meeting At Risk Mental State criteria

**DOI:** 10.1038/s41537-025-00582-5

**Published:** 2025-02-27

**Authors:** Laura J. Bonnett, Alexandra Hunt, Allan Flores, Catrin Tudur Smith, Filippo Varese, Rory Byrne, Heather Law, Marko Milicevic, Rebekah Carney, Sophie Parker, Alison R. Yung, On behalf of the IPPACT Study Group which also includes, Jai Shah, Marita Pruessner, Ashok Malla, Tim Ziermans, Sarah Durston, W. C. Chang, Anthony Morrison, David Shiers, Mark van der Gaag, William McFarlane, Patrick Welsh, Paul Tiffin, Anita Riecher-Rössler, Erich Studerus, Frauke Schultze-Lutter, Stephan Ruhrmann, Joachim Klosterkötter, Suk Kyoon An, Inti Qurashi, Nusrat Huasain, Simon Chu, Paul Amminger, Magdalena Kotlicka-Antczak, Jean Addington, Silvia Rigucci, Swapna Verma, Chun Ting Chan, Masahiro Katsura, Kazunori Matsumoto, Tsutomu Takahashi, Pablo Gaspar, Rolando Castillo, Sebastian Corral, Rocio Mayol-Troncoso, Alejandro Maturana, Peter Uhlhaas, Nicolas Rüsch

**Affiliations:** 1 Department of Health Data Science, University of Liverpool, Liverpool, UK; 2 School of Health Sciences, Division of Psychology & Mental Health, University of Manchester, Manchester, UK; 3 Greater Manchester Mental Health NHS Foundation Trust, Manchester, UK; 4 Institute for Mental and Physical Health and Clinical Translation (IMPACT), Deakin University, Geelong, Australia; 5 Department of Psychiatry, McGill University and Douglas Research Centre, Montreal, Canada; 6 Department of Psychology, University of Utrecht, Utrecht, Netherlands; 7 Department of Psychiatry, School of Clinical Medicine, LKS Faculty of Medicine, University of Hong Kong & State Key Laboratory of Brain and Cognitive Sciences, Hong Kong, China; 8 Faculty of Clinical, Neuropsychological and Developmental Psychology, Amsterdam Public Mental Health research institute, Department of Clinical Psychology, Amsterdam, Netherlands; 9 Tufts University School of Medicine, Maine Health Research Institute, Portland, Maine, USA; 10 Tyne and Wear NHS Foundation Trust and Research Associate, Newcastle University, Cumbria, Northumberland UK; 11 Hull York Medical School & Department of Health Sciences, University of York, Heslington, York, UK; 12 Medical Faculty, University of Basel, Basel, Switzerland; 13 Division of Personality and Developmental Psychology, Department of Psychology, University of Basel, Basel, Switzerland; 14 Department of Psychiatry and Psychotherapy, Medical Faculty, Heinrich-Heine-University, Düsseldorf, Germany; 15 Department of Psychology, Faculty of Psychology, Airlangga University, Surabaya, Indonesia; 16 University Hospital of Child and Adolescent Psychiatry and Psychotherapy, University of Bern, Bern, Switzerland; 17 Department of Psychiatry and Psychotherapy, University of Cologne, Faculty of Medicine and University Hospital of Cologne, Cologne, Germany; 18 Department of Psychiatry, Severance Hospital, Yonsei University College of Medicine, Seoul, South Korea; 19 Mental Health & Neuroscience, University of Liverpool, Liverpool, UK; 20 Psychology, University of Central Lancashire, Lancashire, UK; 21 Orygen, Centre for Youth Mental Health, The University of Melbourne, Parkville, VIC Australia; 22 Early Psychosis Diagnosis and Treatment Lab, Department of Affective and Psychotic Disorders, Medical University of Lodz, Lodz, Poland; 23 Department of Psychiatry, Hotchkiss Brain Institute, University of Calgary, Calgary, Canada; 24 Mental Health Department, ALS, Roma 1, Rome, Italy; 25 Department of Psychosis, Institute of Mental Health, Singapore, Singapore; 26 Canal Kotodai General Mental Clinic, Sendai, Japan; 27 Kokoro no clinic OASIS, Sendai, Japan; 28 Department of Neuropsychiatry, University of Toyama, Graduate School of Medicine and Pharmaceutical Science, Toyama, Japan; 29 Department of Psychiatry, IMHAY, University of Chile, Santiago, Chile; 30 Institute of Neuroscience and Psychology, University of Glasgow, Glasgow, UK; 31 Department of Child and Adolescent Psychiatry, Charité - Universitätsmedizin Berlin, Berlin, Germany; 32 Department of Psychiatry II, Ulm University & BKH Günzburg, Günzburg, Germany

**Keywords:** Psychosis, Biomarkers

## Abstract

**Background:**

The At Risk Mental State (ARMS) (also known as the Ultra or Clinical High Risk) criteria identify individuals at high risk for psychotic disorder. However, there is a need to improve prediction as only about 18% of individuals meeting these criteria develop a psychosis with 12-months. We have developed and internally validated a prediction model using characteristics that could be used in routine practice.

**Methods:**

We conducted a systematic review and individual participant data meta-analysis, followed by focus groups with clinicians and service users to ensure that identified factors were suitable for routine practice. The model was developed using logistic regression with backwards selection and an individual participant dataset. Model performance was evaluated via discrimination and calibration. Bootstrap resampling was used for internal validation.

**Results:**

We received data from 26 studies contributing 3739 individuals; 2909 from 20 of these studies, of whom 359 developed psychosis, were available for model building. Age, functioning, disorders of thought content, perceptual abnormalities, disorganised speech, antipsychotic medication, cognitive behavioural therapy, depression and negative symptoms were associated with transition to psychosis. The final prediction model included disorders of thought content, disorganised speech and functioning. Discrimination of 0.68 (0.5-1 scale; 1=perfect discrimination) and calibration of 0.91 (0-1 scale; 1=perfect calibration) showed the model had fairly good predictive ability.

**Discussion:**

The statistically robust prediction model, built using the largest dataset in the field to date, could be used to guide frequency of monitoring and enable rational use of health resources following assessment of external validity and clinical utility.

## Introduction

Identification of individuals at high risk of developing a first episode of psychosis is possible through use of the Ultra High Risk (UHR) criteria^1–3^, also known as the At Risk Mental State (ARMS) and Clinical High Risk (CHR) criteria^4^. A recent meta-analysis reported transition rates for UHR individuals was 14% at 12 months, 25% at 3 years, and 29% at 4 years. Using the subset of studies that included Kaplan-Meier plots, the estimated transition rate at 10 years was 35%^5^. This demonstrates that UHR individuals have a much higher risk of developing a psychotic disorder than the general population^5^.

There is evidence that the UHR criteria are relatively specific for psychotic disorders. For example, Webb^6^ found that the incidence of psychotic disorder was significantly higher in a UHR sample when compared to help-seeking controls whilst Lin^7,8^ showed that the incidence of new cases of non-psychotic disorders was low compared to the incidence of psychotic disorder in a UHR sample.

There is also evidence that the determination of transition to psychosis, while a necessarily arbitrary point, is clinically meaningful. For example, in a North American study nearly 82% (27/33) of UHR individuals who developed a psychotic disorder met criteria for continued psychosis at 1 year follow up. This proportion was not significantly different from a first episode psychosis sample that was independently recruited^9^. A separate study found that 64% of UHR individuals who developed psychosis met criteria for a current or lifetime psychotic disorder at long term follow up^7^. Consistent with these findings, UHR individuals who develop psychosis have worse functioning compared to those who do not transition at follow up^10^, and show grey and white matter changes around the time of transition to psychosis^11–14^.

Thus, UHR individuals are at substantially higher risk of developing a psychotic disorder than their non-UHR counterparts and development of psychosis is a clinically meaningful outcome. Detection and management of UHR individuals aiming to prevent onset of psychosis is therefore a justifiable goal^1^. Indeed, assessment of the UHR criteria forms part of national clinical assessment in Early Intervention in Psychosis Services in England and Australia^15^. However, most individuals meeting the UHR criteria will not develop a psychotic disorder^16^. This means that some might be receiving unnecessary treatment and health services may be using costly interventions such as Cognitive Behaviour Therapy (CBT) in people who may not need it. Improved prediction of which UHR individuals are at highest and lowest risk would allow clinical trials to target those at highest risk and enable research into the pathophysiology of psychotic disorders by focussing on those most likely to ‘transition’ to psychosis. But of most clinical relevance is that improved identification of those at highest risk of transition would enable busy clinical services to provide more rational resource allocation. To this end, in this study we aimed to (i) synthesise current evidence of predictors of psychosis using an Individual Patient Data (IPD) meta-analysis, and (ii) develop and internally validate a prediction model using measures that are easily applied in routine clinical practice using factors found to be predictive in the IPD meta-analysis. This is the first study in the field to use a large dataset from multiple smaller ones to build a statistically robust prediction model focussing on measures identified from the literature and focus groups that are easily applied in clinical practice.

## Results

### Results of the individual participant data meta-analysis

A PRISMA-style diagram illustrating the flow of studies and participants through the systematic review and meta-analysis can be seen in Section 1 of the Supplementary Material.

**Fig. 1:**
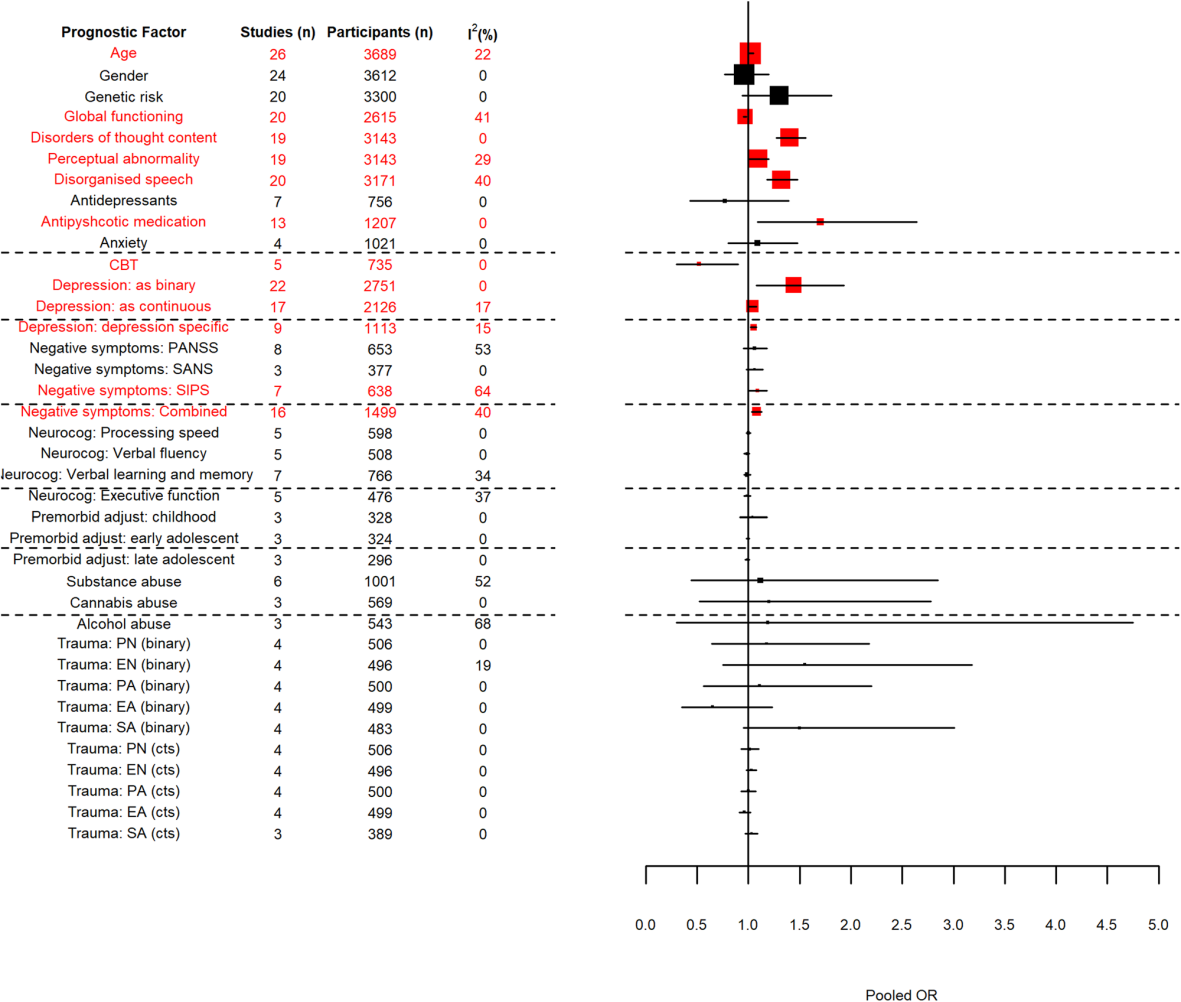
A forest plot showing the results of the IPA-MA per prognostic factor. Factors in red are statistically significant. Boxes are weighted by study size with the largest boxes depicting the largest studies. The dashed lines are included to improve legibility.

The IPD dataset consisted of 3739 participants from 26 studies – CAYR^17^, deWit^18^, DUPS^19^, EASY^20^, EDIE 1^21^, EDIE 2^22^, EDIE-NL^23^, EDIPP^24^, FEPSY^25^, FARMS^26^, FETZ^27^, Grape^28^, NAYAB^29^, NEUROPRO^30^, Norway^31^, PACE^32^, PORT^33^, Predict^34^, SAFE^35^, Rome^36^, SWAP^37^, Tohuku^35^, Toyama University^38^, UCHIP^39^, YouR^40^ and ZinEP^41^. Results for each prognostic factor within the meta-analysis can be seen in Table 1 and Fig. 1. Forest plots for each prognostic factor can be seen Section 3 of the Supplementary Material. The variables statistically associated with transition to psychosis following meta-analysis were age, global functioning score, disorders of thought content, perceptual abnormalities, disorganised speech, antipsychotic medication, CBT, depression (irrespective of analysis approach), and negative symptoms (when considering SIPS only, or the combined negative symptoms score).

**Table 1:** IPD-MA per prognostic factor for transition to psychosis by 12-months (factors in bold were considered for potential inclusion in the prediction model).

Prognostic Factor	Studies	Participants	Pooled estimate	I^2^
	(*n*)	(*n*)	OR (95% CI)	(%)
**Age**	26	3689	1.02 (1.00, 1.05)	22
**Gender**	24	3612	0.96 (0.77, 1.20)	0
**Trait Vulnerability of psychosis**	20	3300	1.30 (0.94, 1.81)	0
**Global functioning score**	20	2615	0.97 (0.95, 0.98)	41
**Disorders of thought content**	19	3143	1.40 (1.27, 1.56)	0
**Perceptual abnormality**	19	3143	1.10 (1.00, 1.20)	29
**Disorganised speech**	20	3171	1.32 (1.18, 1.48)	40
Antidepressants	7	756	0.77 (0.43, 1.39)	0
Antipsychotic medication	13	1207	1.70 (1.09, 2.64)	0
Anxiety	4	1021	1.09 (0.80, 1.48)	0
CBT	5	735	0.52 (0.30, 0.90)	0
Depression				
As binary variable	22	2751	1.44 (1.08, 1.93)	0
As continuous Z-score	17	2126	1.04 (1.01, 1.08)	17
Depression specific metrics	9	1113	1.05 (1.02, 1.08)	15
Negative symptoms				
PANNS	8	653	1.06 (0.95, 1.18)	53
SANS	3	377	1.06 (0.98, 1.14)	0
SIPS	7	638	1.09 (1.01, 1.18)	64
Combined	16	1499	1.08 (1.03, 1.13)	40
Neurocognitive variables				
Processing Speed	5	598	1.00 (0.98, 1.02)	0
Verbal Fluency	5	508	0.99 (0.96, 1.01)	0
Verbal Learning & Memory	7	766	0.99 (0.96, 1.02)	34
Executive Function	5	476	0.99 (0.96, 1.02)	37
Premorbid adjustment*				
Childhood (total)	3	325	1.04 (0.92, 1.18)	0
Early Adolescent (total)	3	324	1.00 (0.98, 1.01)	0
Late Adolescent (total)	3	296	0.99 (0.97, 1.01)	0
Substance abuse				
Substance Abuse	6	1001	1.12 (0.44, 2.85)	52
Cannabis	3	569	1.20 (0.52, 2.78)	0
Alcohol	3	543	1.19 (0.30, 4.75)	68
Trauma				
As binary variables				
Physical Neglect	4	506	1.18 (0.64, 2.18)	0
Emotional Neglect	4	496	1.18 (0.64, 2.18)	19
Physical Abuse	4	500	1.11 (0.56, 2.20)	0
Emotional Abuse	4	499	0.65 (0.35, 1.23)	0
Sexual Abuse	4	483	1.50 (0.95, 3.01)	0
As continuous variables				
Physical Neglect	4	506	1.01 (0.93, 1.10)	0
Emotional Neglect	4	496	1.03 (0.98, 1.08)	0
Physical Abuse	4	500	1.00 (0.93, 1.07)	0
Emotional Abuse	4	499	0.96 (0.91, 1.02)	0
Sexual Abuse	3	389	1.03 (0.97, 1.09)	0

*Results for the total per age group are presented here. Results for the scores per subscale per age group are available in Section 3 of the Supplementary Material.

### Results of the focus groups

In general, service users easily reached consensus regarding acceptable variables but were keen to ensure that trauma assessments were sensitively made with follow-up wellbeing checks as standard. Staff also reached consensus regarding acceptable variables but had concerns about the feasibility in routine practice of delivering a full assessment battery due to time constraints within a standard appointment. There was also recognition of an ethnic/cultural disparity in presentations to ARMS specialists vs EIP or Crisis. This may suggest that stigma and/or fear of services has a heavier impact among some ethnic/cultural minority groups and prevents help-seeking until crisis-point. Therefore, there was a suggestion to include ethnicity data where possible.

Overall, all of the factors identified through the meta-analysis as statistically associated with transition to psychosis were deemed acceptable and feasible for routine assessment at clinical services by both service users and staff. Consequently, no factors were removed from the pool of factors considered for potential inclusion in the prediction model.

### Results of model development and testing

The IPD dataset for the prediction modelling consisted of 2909 people meeting ARMS criteria from 20 studies – CAYR^17^, DUPS^19^, EASY^20^, EDIE 2^22^, EDIE-NL^23^, EDIPP^24^, FEPSY^25^, FARMS^26^, FETZ^27^, Grape^28^, NAYAB^29^, NEUROPRO^30^, PACE^32^, PORT^33^, SAFE^35^, SWAP^37^, Toyama University^38^, UCHIP^39^, YouR^40^ and ZinEP^41^. EDIE2, EDIE-NL, NAYAB and NEUROPRO are randomised controlled trials. Table 2 provides a demographic summary of the individuals used in the prediction modelling together with negative symptom scores which were of clinical interest but contained too much missing data to be used within the modelling process.

**Table 2:** Demographic summary of people at baseline in the IPD dataset used for the prediction modelling – numbers are median (interquartile range) [range] unless otherwise stated.

Variable	Did not transition	Transitioned	Total
	(*n* = 2550)	(*n* = 359)	(*n* = 2909)
Gender, *n* (%)			
Male	1385 (55)	204 (57)	1589 (55)
Female	1155 (45)	155 (43)	1310 (45)
*Missing*	*10 (0)*	*0 (0)*	*10 (0)*
Trait Vulnerability, n (%)			
No	2210 (87)	307 (86)	2517 (87)
Yes	340 (13)	52 (14)	392 (13)
Age (years)	19 (17, 23)	20 (17, 24)	20 (17, 23)
	[12–51]	[12–39]	[12–51]
			*26*
Combined Score: Disorders of thought content	4 (3, 5)	4 (3, 5)	4 (3, 5)
	[0 to 6]	[0–6]	[0–6]
			*38*
Combined Score: Perceptual abnormalities	3 (2, 4)	4 (2, 4)	3 (2, 4)
	[0–6]	[0–6]	[0–6]
			*38*
Combined Score: Disorganised speech	2 (0, 3)	2 (0, 4)	2 (0, 3)
	[0–6]	[0–6]	[0–6]
			*40*
Global Functioning Scale, mean (standard deviation) [range]	53 (13)	47 (14)	51 (13)
	[1–96]	[18–91]	[1–96]
			*586*
Treated. *n* (%)			
No	147 (6)	13 (4)	160 (6)
Yes	589 (23)	76 (21)	665 (22)
Not known	1814 (71)	270 (75)	2084 (72)
Negative symptom scores			
PANSS (total)	7·0 (3·0, 12·0)	7·0 (3·0, 12·0)	7·0 (3·0, 12·0)
	[1.0, 36.0]	[1.0, 36.0]	[1.0, 36.0]
			*2364*
SANS (total)	3.0 (0.75, 7.0)	4·0 (1.0, 8.0)	3.0 (1.0, 7.00)
	[0.0, 17.0]	[0.0, 16.0]	[0.0, 17.0]
			*2556*
SIPS (total), mean (standard deviation)	12.8 (6.1)	12.8 (6.1)	12.8 (6.1)
	[0.0, 29.0]	[0.0, 29.0]	[0.0, 29.0]
			*2579*

Missing values are in italics.

A demographic summary of people per dataset can be seen in Section 4 of the Supplementary Material– this does not include negative symptom scores due to the extensive missingness.

The parsimonious multivariable model (after backwards selection) for transition to psychosis included disorders of thought content, disorganised speech, global functioning score, and treatment (which was forced into the model). All three predictors were statistically associated with the outcome. The optimism-adjusted c-statistic for the model was 0.68 and the calibration slope, adjusted for model fitting optimism, was 0.91. These show fairly good discrimination and calibration. Optimism adjusted model estimates can be seen in Table 3. Unadjusted estimates for all analyses can be found in Section 5 of the Supplementary Material.

**Table 3:** Parsimonious multivariable model for risk of transition to psychosis according to the IPD dataset (adjusted for shrinkage).

Variable	Odds Ratio
	(95% CI)
*Intercept, log odds ratio (standard error)*	*3.13 (0*.*61)*
Combined score: Disorders of thought content (linear)	1.32 (1.20, 1.45)
Combined score: Disorganised speech (linear)	1.21 (1.12, 1.30)
Global Functioning Scale (linear)	0.97 (0.96, 0.98)
Treatment	
No	1.00
Yes	0.83 (0.44, 1.58)
Not known	0.98 (0.47, 2.02)

The intercept, shown in italics, is a log odds ratio rather than an odds ratio.

Odds ratio > 1 implies risk of transition to psychosis is greater in this group than in the baseline group

According to the fractional polynomial approach, the functional form for each continuous variable was linear. Therefore, for each unit increase in disorders of thought content score there was a 32% increase in risk of transitioning to psychosis while for each unit increase in disorganised speech score there was a 21% increase in risk, after adjusting for other covariates. Results for the global functioning scale show that for each unit increase in the scale, there is an associated 3% decrease in risk of transitioning to psychosis. Therefore, disorders of thought content has the highest predictive value within the model.

Based on the results shown in Table 3, the full prediction model can be written as shown in Eq. 1.

1 $${\mathrm{ln}}\left(\frac{{p}_{i}}{1-{p}_{i}}\right)=3.13+(0.28\times DT{C}_{i})+(0.19\times D{S}_{i})+(-0.03\times GA{F}_{i})+\,(-0.19\times Treat{Y}_{i})+(0.02\times TreatN{K}_{i})$$

where *p*_*i*_ is the probability of transitioning to psychosis by 12 months, $\mathrm{ln}(\frac{{p}_{i}}{1-{p}_{i}})$ is the log odds of the outcome, *DTC* is disorders of thought content, *DS* is disorganised speech, *GAF* is Global Functioning Scale, *TreatY* is yes to treatment, *TreatNK* is unknown treatment status, and *i* is each individual in the dataset. For all variables, yes is coded as 1 and no is coded as 0. Risk (outcome probability) predictions, *p̂*, for a new individual can be estimated by inputting their predictor values into the equation and then transforming back to the probability scale as shown in Eq. 2.

2 $$\hat{p}=\frac{\exp (L{P}_{i})}{1+\exp (L{P}_{i})}$$

where:

$$L{P}_{i}=3.13+(0.28\times DT{C}_{i})+(0.19\times D{S}_{i})+(-0.03\times GA{F}_{i})+\,(-0.19\times Treat{Y}_{i})+(0.02\times TreatN{K}_{i})$$

#### Sensitivity analyses

##### Attenuated psychotic symptoms

Demographic data for the APS group can be found in Section 6 of the Supplementary Material. The parsimonious multivariable model, with genetic risk replaced with APS as a potential predictive factor, included the same covariates as the original model: disorders of thought content, disorganised speech and functioning as well as treatment which was forced into the model. Being a member of the APS group was not included in the model. Results are as described in section 3.2 and in Table 3.

##### Omega-3 fatty acids

Demographic data for omega-3 fatty acids being included as part of the treatment variable can be found in Section 6 of the Supplementary Material. The parsimonious multivariable model for transition to psychosis, with people taking omega-3 fatty acids coded as receiving treatment, included the same covariates as the original model. The optimism-adjusted c-statistic for this model was 0.68 and the calibration slope, adjusted for model fitting optimism, was 0.91 as for the original model. Again, these measures show fairly good discrimination and calibration. Optimism adjusted model estimates can be seen in Table 4. Unadjusted estimates for all analyses can be found in Section 6 of the Supplementary Material. The direction of effect is consistent with the original model showing that the recoding of omega-3 as a treatment had little effect on the prediction model.

**Table 4:** Parsimonious multivariable model for risk of transition to psychosis according to the IPD dataset (adjusted for shrinkage) with people taking omega-3 coded as treated.

Variable	Odds Ratio
	(95% CI)
*Intercept, log odds ratio (standard error)*	−*3*.*19 (0*.*61)*
Combined score: Disorders of thought content (linear)	1.32 (1.19, 1.45)
Combined score: Disorganised speech (linear)	1.21 (1.12, 1.30)
Global Functioning Scale (linear)	0.97 (0.96, 0.98)
Treatment	
No	1.00
Yes	0.82 (0.43, 1.57)
Not known	1.02 (0.50, 2.07)

The intercept, shown in italics, is a log odds ratio rather than an odds ratio.

Odds ratio > 1 implies risk of transition to psychosis is greater in this group than in the baseline group

##### Cohort studies

The parsimonious multivariable model based on only the cohort studies within the IPD dataset, included the combined score for disorders of thought content, the combined score for disorganised speech, global functioning score, presence of trait vulnerability and treatment (which was forced into the model). Being a member of the trait vulnerability group is an additional variable not included in the original model. The optimism-adjusted c-statistic for the model was 0.67 and the calibration slope, adjusted for model fitting optimism, was 0.91 which are very similar to those for the original model, and thus show fairly good model performance. Optimism adjusted model estimates can be seen in Table 5. Unadjusted estimates for all analyses can be found in Section 6 of the Supplementary Material. The results are in the same direction as the original model for disorganised thought content, disorganised speech, and global functioning scale. The results for trait vulnerability suggest that people with such a risk are 51% more likely to transition to psychosis than those without, although this predictor is not significantly associated with the outcome when accounting for the other predictors.

**Table 5:** Parsimonious multivariable model for risk of transition to psychosis according to the cohort studies within the IPD dataset (adjusted for shrinkage).

Variable	Odds Ratio
	(95% CI)
*Intercept, log odds ratio (standard error)*	−*3*.*84 (0*.*77)*
Combined score: Disorganised thought content (linear)	1.33 (1.18, 1.48)
Combined score: Disorganised speech (linear)	1.28 (1.17, 1.41)
Global Functioning Scale (linear)	0.98 (0.96, 0.99)
Trait Vulnerability	
No	1.00
Yes	1.51 (0.99, 2.30)
Treatment	
No	1.00
Yes	1.63 (0.58, 4.56)
Not known	1.45 (0.49, 4.28)

The intercept, shown in italics, is a log odds ratio rather than an odds ratio.

Odds ratio > 1 implies risk of transition to psychosis is greater in this group than in the baseline group

##### Global functioning score

The three studies which did not provide data related to global functioning led to a parsimonious model which included the combined score for disorders of thought content, the combined score for disorganised speech, global functioning score, presence of trait vulnerability and treatment (which was forced into the model). Presence of trait vulnerability is an additional variable not observed in the original model. The optimism-adjusted c-statistic for the model was 0.69 and the calibration slope, adjusted for model fitting optimism, was 0.91 which are again very similar to the original model, and thus show fairly good model performance. Optimism adjusted model estimates can be seen in Table 6. Unadjusted estimates for all analyses can be found in Section 6 of the Supplementary Material. The results are in the same direction as the original model for disorganised thought content, disorganised speech, and global functioning scale. Once again, trait vulnerability is not significantly associated with the outcome when adjusting for the other predictors but does suggest that people with trait vulnerability have a higher chance of transitioning to psychosis than those without.

**Table 6:** Parsimonious multivariable model for risk of transition to psychosis according to the cohort studies within the IPD dataset (adjusted for shrinkage).

Variable	Odds Ratio
	(95% CI)
*Intercept, log odds ratio (standard error)*	−*3*.*24 (0*.*62)*
Combined score: Disorganised thought content (linear)	1.33 (1.20, 1.48)
Combined score: Disorganised speech (linear)	1.25 (1.15, 1.35)
Global Functioning Scale (linear)	0.97 (0.96, 0.98)
Trait Vulnerability	
No	1.00
Yes	1.36 (0.97, 1.92)
Treatment	
No	1.00
Yes	0.82 (0.43, 1.55)
Not known	0.95 (0.46, 1.97)

The intercept, shown in italics, is a log odds ratio rather than an odds ratio.

Odds ratio > 1 implies risk of transition to psychosis is greater in this group than in the baseline group

## Discussion

The UHR criteria are associated with risk of developing a psychotic disorder. However, there is a need to identify which UHR individuals are at higher risk of transition and which are at lower risk. To examine this issue, we conducted an individual participant data meta-analysis and then developed and internally validated a clinical prediction model. We focussed on characteristics that could be used in routine practice in order to create a clinically relevant prediction tool. This systematic review and individual participant data meta-analysis pooled prognostic factors from 26 studies (3739 individuals) to develop a clinical prediction model for psychosis risk in ARMS individuals. The final model included thought content disorders, disorganised speech, global functioning score, and treatment (forced into the model). It demonstrated fairly good predictive performance, with an optimism-adjusted c-statistic of 0.68 and a calibration slope of 0.91.

Within the individual participant data meta-analysis, higher age, disorders of thought content, perceptual abnormalities, disorganised speech, antipsychotic medication, depression and negative symptoms were all significantly associated with higher risk of developing psychotic disorders, and higher global functioning and cognitive behavioural therapy demonstrated a significantly lower risk.

The finding that high levels of subthreshold positive psychotic symptoms increases risk of transition is not surprising, given that the definition of transition is based on positive symptoms. Thus, for example, a shift from holding some doubt about the malicious intentions of others to believing 100% that others are planning to hurt one represents the onset of psychotic disorder. The risk associated with higher age may indicate that the individual has been experiencing symptoms for a longer period. Longer duration of UHR symptoms prior to seeking help was a risk factor to transition in one previous large study^42^.

CBT was found to be associated with lower risk of transition. This may be due to the protective effect of CBT, and there is some evidence for this^43^. It could also be that those who are able to access this treatment may have higher cognitive functioning and fewer negative symptoms than those not accessing CBT. Conversely, our finding that use of antipsychotics increased risk of psychosis may be because those antipsychotics were prescribed to those with high levels of symptoms or those that a clinician thought were close to transition or at highest risk of transition.

Negative symptoms were a risk factor for transition. This may be because individuals with high levels of negative symptoms may be developing a chronic psychotic illness such as schizophrenia. In support of this, high negative symptoms is associated with lack of clinical and functional recovery, even in early intervention services^44^.

All of the above variables that were significantly associated with risk of developing a psychotic disorder were considered suitable for use in routine clinical practice by consumers and staff members. The final prediction model included disorders of thought content, disorganised speech and global function. Negative symptoms had to be excluded from the final model due to a large amount of missing data. The included variables are similar to some predictors in the risk prediction tool developed by Cannon et al., the NAPLS-2 Risk calculator, which included a summed score of unusual thought content and suspiciousness and decline in social functioning^45^. These were significant predictors of psychosis and were included in Cannon et al.’s final model^45^. Unlike Cannon et al., who found that verbal learning and memory significantly predicted transition, no cognitive variables were significant predictors of psychosis in our study.

Discrimination of the model was fairly good at 0.68 and similarly calibration, at 0.91 after adjusting for model optimism. These values are similar to Cannon et al.’s model^45^ which included the following features: a summed score of unusual thought content and suspiciousness, decline in social functioning, a family history of psychosis, verbal learning and processing speed, age, history of trauma and stressful life events. Their overall model achieved a C-index of 0.71. Whilst benchmarks do not currently exist for model performance measures within clinical areas, anecdotal evidence suggests that the c-statistic can range from 0.6 (epilepsy prediction models) to 0.9 or more (pancreatic cancer models). The NAPLS-2 model was externally validated^46^ in 210 subjects from the Early Detection, Intervention, and Prevention of Psychosis Program^24^ and showed good discrimination, with an AUC of 0.790 (95% CI = 0.644-0.937), although in this validation study the researchers did not include trauma and stress life events in their model. The justification for this was that these variables were not significant predictors of psychosis in the original Cannon study^45^. However, this means that they did not actually evaluate the model as originally described. A further external validation study was carried out at the Shanghai At Risk for Psychosis Program (SHARP) in 200 UHR individuals^47^. This study also omitted trauma and stress life events. The resulting AUC value was 0.631 (95% CI 0.542–0.721). Thus the NAPLS-2 risk calculator did not fit this data set as well as it did the original sample or the first validation sample, perhaps because the population was different (original study and first validation both carried out in USA, SHARP study in China) or sample ascertainment, treatment, or other factors were different.

The current study has limitations. The use of IPD maximises the event rate within the dataset, however, the IPD dataset included data from randomised trials and cohort studies. These different sources could have had effects on the risk of transition, and potentially on the model, as seen by the inclusion of trait vulnerability in the model built using only data from cohort studies. The inclusion of individuals who were prescribed antipsychotic medication is a potential confound. Also, there is a high percentage of missing data within the IPD dataset which was accounted for by multiple imputation. This means that the model performs artificially well in the development data. This would be even higher if variable with greater than 50% missingness had been included in the model. In the related sensitivity analysis, the exclusion of datasets with large amounts of missing data led to the inclusion of trait vulnerability in the final model, although this was not significantly associated with the outcome when adjusting for other factors. It would be of interest to also evaluate the impact of varying the cut-offs used for the anxiety scores as this may have increased the predictive power of this variable, potentially removing another variable from the model.

Another limitation is that most of the included studies were from Western developed areas and so the model may not perform as well in services based in developing nations. Indeed, the need to have consistency between datasets for the meta-analysis and systematic review led to additional limitations. For example, most studies did not report frequency of use or volume of substance used for individuals reporting substance abuse. Therefore, a simple dichotomous variable for substance abuse was the only option removing the ability to fully evaluate the level of substance abuse associated with transitioning to psychosis. Also, there was a need to cojoin disorders of thought content with suspiciousness. Again this limits the ability to evaluate the impact of each aspect separately on the outcome, but is in line with other studies in the area^45,48^.

Further work is now required to validate the model in independent data. This is being done in related research by the authors (UK National Institute for Health Research HTA Project 17/31/05). This related project is recruiting a cohort study with at least 100 ARMS individuals, from across the UK, developing a psychotic disorder within 12 months^49^ to determine the validity of the developed model. Ultimately, this will enable the identification of a sub-group of patients at low risk of transition in whom it is considered safe to undergo regular mental state monitoring, and, in contrast, to identify a sub-group of patients at high risk of transition to psychosis who will need more intensive support from services and access to rapid specific treatment should they develop a psychotic disorder. Such stratification of care pathways will allow more rational allocation of health services resources, more appropriate appointment management, and could lead to improved patient outcomes. Following external validation, further work is needed to determine the model’s clinical utility. For example, we need to determine whether or not clinicians would use such a tool to assist in their management of a UHR patient, and to assess whether UHR individuals with low risk of transition are offered monitoring and whether those at higher risk are offered more extensive intervention such as CBT. A health economic evaluation of such stratification of care pathways would assist policy makers in assessing the benefits (or otherwise) of such an approach. The final stage of the work would then be to integrate the model into existing clinical workflows via NHS Local Champions and wider rollout.

### Conclusion

Use of a clinical prediction model for risk of transition to psychosis in people meeting UHR criteria has the potential to improve treatment decisions, reduce unnecessary interventions, and maximise the use of resources. The model developed here has been shown to perform well in a dataset comprising data from 20 studies. The next steps include testing the validity of the model in independent data (external validation), discussing the most appropriate presentation method for this model with end-users, testing the clinical utility of the model within clinical practice, evaluating the health economics of the model, and ensuring that this model is adopted as part of routine clinical practice to improve the lives of UHR individuals.

## Methods

### Individual patient data meta-analysis

We chose to utilise an individual Patient Data (IPD) meta-analysis rather than using aggregated data extracted from research reports. The IPD meta-analysis method evaluates and reanalyses individual-level data from all studies addressing a particular clinical research question. IPD meta-analyses are able to exploit a larger combined sample size than individual primary studies and can test their generalisability across different settings and populations. This approach is particularly informative when reviewing complex clinical areas with the aim of exploring variation and predictors of variation in outcomes. IPD meta-analysis can also standardise the statistical analyses in each study, and estimate the prognostic factor results directly from the IPD of each study, independent of study reporting quality and the significance of the findings, therefore decreasing risk of publication/selection bias. This method also has the ability to check modelling assumptions and perform adjusted analyses in each study with a consistent set of adjustment factors, thus examining interactions between prognostic factors.

#### Database assembly

##### Population

We assembled a dataset for development of psychotic disorder in UHR patients from multiple studies including individuals meeting UHR criteria. These are defined as 1) attenuated psychotic symptoms (APS), 2) full-blown brief limited intermittent psychotic symptoms (BLIPS) and/or 3) genetic/familial risk for schizophrenia in conjunction with a significant decrease in functioning. These groups are operationalised using measures such as the Comprehensive Assessment of At-Risk Mental States (CAARMS)^50^ or the Structured Interview for Prodromal Syndromes (SIPS)^51^. Included studies must have been published in 1994 or more recently to be included in the dataset, as 1994 was the initial year of the first prospective study using ARMS criteria^1,52^. Only studies meeting the defined ARMS criteria were included.

##### Study design

Any prospective study (i.e., cohort studies and randomised controlled trials of preventive interventions) with participants meeting the above-mentioned criteria were eligible. Studies needed to include a baseline assessment, at least 12-month follow-up longitudinal assessments, and the assessment of transition to psychosis in UHR individuals.

##### Outcomes

The main outcome measures was the development of psychotic disorder at 12-months. This was defined using standard diagnostic classification systems (Diagnostic and Statistical Manual of Mental Disorders-III, IV-TR, and 5 (DSM), and International Classification of Diseases-10 (ICD-10))^53–56^ or commonly used UHR assessment schedules (e.g., SIPS or CAARMS). The 12-month point was chosen as this is the highest risk period for psychosis onset^32^, the time when individuals are most distressed, and the time they are most likely to engage with services^43^.

##### Identification of studies

We conducted a literature search using CINAHL, EMBASE, PubMed and PsychInfo. The searches were supplemented by inspection of studies included in previous systematic reviews and meta-analyses of psychotic transition studies, inspection of reference lists of psychosis transition studies identified through database searches, and inspection of citations of psychosis transition studies identified through database searches. No restrictions were placed on the language of the publication. Titles, abstracts, and keywords were searched in the publication databases using search terms adapted from previous systematic reviews and meta-analyses of this research area: [‘psychosis’] AND [‘clinically at high risk’ OR ‘clinically at risk’ OR ‘clinical high risk’ OR ‘ultra-high risk’ OR prodrom* OR ‘at risk mental state’ OR ‘risk of psychosis’ OR ‘’ OR ‘ARMS’ OR ‘prodromal psychosis’]. A combination of the four strings was used to scope out searches with respect to symptom and outcome of psychosis:

1. At risk mental state OR basic symptoms OR prodromal psychosis OR psychosis risk OR UHR OR ultra high risk OR clinical high risk OR CHR
2. Prodrom* AND psychosis*
3. ARMS AND Psychosis
4. High risk AND Psychosis*

The final list of included studies was supplemented by knowledge of lead investigators and collaborators to ensure all possible relevant studies were included. Further details of the review can be found in the associated publication^57^.

##### Data collection & database development

Contact information for authors of eligible studies were identified from the published trials. An initial email was sent to the corresponding author, providing them a summary protocol for the overall study. Another investigator from the study was contacted if initial email failed to receive a response.

We derived a set of pre-specified and defined variables at both the outcome, participant and trial level and shared this with authors of relevant studies. In particular, the following data was requested for all participants: baseline data for narrative purposes, details of any intervention(s), and the outcome. Authors of eligible studies provided de-identified data in any format. This was recoded as required, verified, and checked for consistency with published data. Where inconsistencies were identified, discussions were held with individual study groups to attempt to resolve these.

#### Individual Participant Data Meta-Analysis (IPD-MA)

We examined the contribution of prognostic factors for transition to psychosis at 12-months via a series of random effects IPD-MAs. For each prognostic factor, a two-step IPD meta-analysis was used to obtain the pooled effect. This approach first estimates prognostic effects from the IPD in each study separately, and then pools them using a conventional meta-analysis. A random effects approach was employed, and statistical heterogeneity was examined using the ‘tau-squared’ statistics (which provides an estimate of between-study variance) and I2 (which provides the proportion of total variance that is due to between-study variance). In many cases, it was necessary to make decisions to standardise variables across datasets. Those decisions are described per variable as follows.

#### Positive symptoms

##### Disorders of thought content

Total disorders of thought content was the highest score on the BPRS Suspiciousness or BPRS Unusual Thought Content items, or the PANSS Suspiciousness/persecution item converted to a CAARMS score on the Disorders of Thought Content scale, or the SIPS Unusual thought content/Delusional ideas score.

##### Perceptual abnormalities

Total perceptual abnormalities score was the highest score on the BPRS Hallucinations item or the PANSS Hallucinatory behaviour item converted to a CAARMS Perceptual Abnormalities score, or the SIPS Perceptual abnormalities/Hallucinations score.

##### Disorganised speech or communication

Total disorganised speech score was the highest score on the BPRS Conceptual disorganisation item or PANSS Conceptual disorganisation item converted to CAARMS Disorganised speech score.

##### Negative symptoms

Total negative symptoms using the Positive and Negative Syndrome Scale (PANSS) was the sum of the response to each of the seven negative subscores (N1-7), or, in the case of the Früherkennung von Psychosen (FEPSY) study, the sum of the response to each of the negative sub-scores on the Brief Psychiatric Rating Scale (BPRS) (16-18)^58,59^. Schedule for the Assessment of Negative Symptoms (SANS) scores were the sum of the score for subscales for global rating of affective flattening (subscale 8), global rating of alogia (subscale 13), global rating of avolition/apathy (subscale 17) and global rating of anhedonia/asociality (subscale 22). Total negative symptoms according to SIPS was the sum of the response to each of the six negative sub-scores (N1-6). Measures were considered on their own but also as a combined variable. In the situation where a study reported multiple measures, SIPS was chosen as the primary measure for inclusion in the combined scores.

##### Global functioning

The GAF and the SOFAS range from 1 to 100 and are divided into 10-point intervals describing the level of functioning and symptoms. The anchor point of 1–10 describes the most severely ill (persistent inability) and the anchor point of 91–100 describes the healthiest (superior functioning). The scores are strongly correlated with evidence showing Spearman correlation coefficients ranging between 0.86 and 0.93 for the comparison of the total scores at baseline and at months 6, 12, 18 and 24 (all *p*-Values < 0.0001)^60^. As most of our datasets (*n* = 16, 62%) provided GAF, we converted the studies the data from studies with SOFAS to GAF. The conversion followed the methodology of Samara et al.^60^ and illustrated in the section “Results of the individual participant data meta-analysis” of the Supplementary Material.

##### Anxiety

In most cases, anxiety was measured using the Positive and Negative Symptoms Scale (PANSS)^61^ anxiety (G2) scale, and was determined to be present with a rating of mild or more (grade 2–7). For those without the PANSS we used response of mild or higher (1–4) for anxiety psychic or anxiety somatic on the Hamilton Depression Scale (HAMD)^62^.

##### Depression

Data were available in binary and continuous forms across various scales – the Beck Depression Inventory (BDI)^63^, HAMD^62^, Montgomery Asberg Depression Rating Scale (MADRS)^64^, and PANSS^61^. For studies with continuous or ordinal depression a binary variable was created to ensure pooling of results across all studies with depression data. This binary depression variable was created with respect to each study’s s depression rating system that equated to a moderate amount of depression. The threshold for creating a binary depression variable for the BDI, MADRS, and HAMD were established based on established literature for said scales on what equated a moderate amount of depression (^65–67^ respectively) as follows – BDI, 21 or greater; MADRS, 18 or greater; HAMD, 20 or greater; CAARMS, four or greater; PANSS general, four or greater. For the CAARMS and PANSS which are not specific depression tools but contain a depression rating component, staff clinical psychologists were consulted for appropriate ways of converting those to a depression variable. Where appropriate, depression was also converted to Z-scores per scale to create a harmonised depression variable on a continuous scale. This excluded data from the Mini-International Neuropsychiatric Interview (MINI) and comorbid variables as they were only submitted as binary variables. The dose-response effect of depression severity was also examined with only depression specific metrics – PANSS and CAARMS were removed. Although these have one question specifically tailored for depression, they are not standard depression specific questionnaires.

##### Trait vulnerability

A person was classed as having trait vulnerability if they had a family history of a first degree relative with a psychotic disorder or they had a schizotypal personality disorder either in isolation, with attenuated psychosis syndrome, with brief limited intermittent psychotic symptoms (BLIPS), or with both attenuated psychosis syndrome and BLIPS.

##### Neurocognitive variables

Processing speed was assessed via Trail Making Test A^68^ completion time (in seconds). Verbal fluency was assessed in a variety of ways across the studies – most used the Controlled Oral Word Association Test (COWAT F + A + S)^69^, whilst the University of Chile High-risk Intervention Program (UCHIP) considered category fluency: animal naming. The Dutch Prediction of Psychosis Study (DUPS)^19^ was not eligible for the analysis as it only considered COWAT S. The California Verbal Learning Test^70^ was the most frequently used test for verbal learning and memory. Otherwise, studies used the Rey Auditory Verbal Learning Test trial 1-5 total correct, Rey Auditory Verbal Learning Test^71^ trial 1–5 total correct or Hopkins Verbal Learning Test^72^ correct score. No adaptions were required as these scores were felt to be similar. Finally, executive function was usually assessed via the Wisconsin Card Sorting Task (WCST)^73^ preservative error. Otherwise, the WCST Percentage Error was used.

##### Premorbid Adjustment Scale (PAS)

The Pre-Morbid Adjustment Scale (PAS)^74^ is a rating scale that is used to evaluate different aspects of childhood development at four distinct periods: early childhood (age 6 up to age 11), early adolescence (age 12 up to age 15), late adolescence (age 16 to age 18), and adulthood (over 18 years). PAS is graded from zero to six per developmental area, with higher scores indicating increasing difficulties in adjustment.

##### Substance abuse

This variable considered any current (at the time of baseline assessment) use of illicit substances including cannabis and alcohol but excluding nicotine and caffeine.

##### Trauma

The trauma data is measured by the Adverse Childhood Experiences Survey (ACES)^75^, the Childhood Trauma Questionnaire (CTQ)^76^, and the Trauma and Distress Scale (TADS)^77^. Trauma was analysed both as binary predictors for presence of trauma and on a continuous scale for its relationship with risk of development of a psychotic disorder. Analyses for trauma were separated by sub-trauma categories: physical neglect, physical abuse, emotional neglect, emotional abuse, and sexual abuse. Where appropriate, certain questions were inverted to properly reflect the direction of effect of trauma^78,79^.

When analysed as a binary variable, presence of trauma was dichotomized as present or absent based on each scale’s available scoring threshold per sub-trauma. For the CTQ, trauma was categorized as present based on a moderate score as follows: emotional abuse (13 or greater), physical abuse (10 or greater), sexual abuse (8 or greater), emotional neglect (15 or greater), and physical neglect (10 or greater)^79^. Trauma in the TADS was categorized as present for any score greater than or equal to 2 in any sub-trauma category^78^. The ACES did not have a scoring threshold, so a study team clinical psychologist with expertise in trauma (FV), was consulted for appropriate ways to categorize the presence of trauma. It was agreed that scoring as per TADS was most appropriate, except sexual abuse which only was scored as present or absent for each sub-category within ACES.

Trauma was also analysed from available continuous variables to check for a dose-response relationship of exposure to trauma and development of psychotic disorder. The scores were converted to Z-scores to create harmonised sub-trauma variables. This was not possible for ACES sexual abuse which was scored as present or absent only. Therefore, any study reporting sexual abuse via ACES could only be included in the meta-analysis where sexual abuse was included as a binary variable.

##### Treatments

A person was deemed to be on psychotropic medication if they had been issued with a prescription for antidepressant and/or antipsychotic medication. A person was deemed to have received cognitive behavioural therapy (CBT) if they were enrolled in the CBT treatment arm of a clinical trial.

### Focus groups

The aim of this part of the study was to develop a prognostic tool that could be used in routine clinical practice. To ensure that the risk factors selected were feasible and acceptable to clinicians and consumers, we conducted focus groups to discuss possible measures. This is an essential step to ensure successful implementation of a prediction model in clinical practice^80^.

Three consumer focus groups with five participants per group were conducted in the English cities of Manchester, Birmingham and Gateshead. Two NHS staff (managers and clinicians) focus groups with 5–8 participants per group were conducted in Birmingham and Gateshead. All focus groups were audio-recorded and common themes identified. At these groups, the pool of risk factors identified in the IPD MA was presented to gauge opinion about their use in the final prediction model. Any measures thought to be too difficult to assess or arrange in routine practice or that were unacceptable to service users were not included in the pool of factors used for model development.

### Model development & internal validation

Analysis used logistic regression modelling and assumptions of the modelling technique were checked for appropriateness including multicollinearity between risk factors. Cases of collinearity were managed by including only the most clinically meaningful of each interacting pair. Ultimately the prognostic factors for potential inclusion in the model were age, gender, trait vulnerability, global functioning, disorders of thought content, perceptual abnormalities, and disorganised speech. Due to extensive (greater than 50%) missing data, negative symptoms were removed from the model^81^. These prognostic factors were identified based on the systematic review described in section, “Introduction”, and the focus groups described in “Results of the individual participant data meta-analysis”.

Scores disorders of thought content, perceptual abnormalities, and disorganised speech were obtained by harmonising scores from CAARMS, SIPS, PANSS and BPRS following clinical guidance by the co-authors and existing evidence^82^. Whilst UHR status is a frequently reported risk factor for transition to psychosis, it is highly correlated with CAARMS positive symptom scores and therefore only the trait vulnerability component of this measure was considered for potential inclusion in the model.

Any treatment (psychotherapy and/or antidepressants and/or antipsychotics and/or other psychiatric medication) was forced into the model as many studies were randomised controlled trials where all participants received a treatment or placebo^49^. Studies without the treatment variable had an extra category coded for ‘not known’.

Given the IPD dataset used for model development, a one-step meta-analysis approach was taken to account for clustering, where the IPD from all studies were analysed together but with clustering by study accounted for using a dummy variable for study^83^.

Missing data was prevalent in the dataset and thus multiple imputation via chained equations was used; variables with more than 50% missingness were excluded from the model to ensure that most of the variable was reported rather than imputed. Twenty imputed datasets were created using predictive mean matching^84^. All continuous covariates were modelled as such to avoid reducing the power to detect relationships and a loss of predictor information which can arise with categorisation^85^. The combined scores are measured on a scale from zero to six, which is considered a continuous scale from a clinical point of view^86^ and thus modelled as such in this manuscript. The functional form for continuous variables was assessed using fractional polynomial transformations within each imputed dataset^87–90^. Variables were selected for inclusion in the final model within each imputed dataset via backwards selection with a p-value of 0.10. Variables selected for the final model were those that featured in at least 10 of the 20 imputed models.

Apparent measures of model performance were calculated for the final multiply imputed model. The performance of the model was evaluated in terms of discrimination and calibration. Discrimination indicates how well the model separates between individuals who transition to psychosis from those that do not^91^. This was measured using the median Harrell’s c-statistic over the imputed datasets^92^. A c-statistic of 0.5 indicates no discrimination beyond chance, whereas a c-statistic of one indicates perfect discrimination. Calibration assesses if the predicted risk of transition agrees with the observed risk. This was reported graphically using calibration plots, within deciles of risk, for each imputed dataset^93^.

Non-parametric bootstrapping estimated optimism and examined model stability. In each of 500 bootstrap samples, the entire modelling process, including predictor selection, was repeated and the apparent model performance (calibration and discrimination in the bootstrap sample) was compared with the performance in the original sample per multiply imputed dataset. The median optimism across all imputed samples was then used to calculate the optimism-adjusted c-statistic and optimism-adjusted calibration slope^94^. Using the latter as a uniform shrinkage factor, all the predictor effects in the final developed model were penalised in order to account for over-fitting^95^. The pool of potential predictors for the backwards selection in this process was any predictor in a final multivariable model for each imputed dataset.

#### Sensitivity analyses

Four sensitivity analyses were undertaken. In the first, given the clinical interest in UHR status and that 89% of individuals in our IPD dataset met the Attenuated Psychotic Symptoms (APS) UHR criterion, we undertook a sensitivity analysis replacing membership of the trait vulnerability group with membership of the APS group as a potential predictive factor. In the second, anyone treated with omega-3 fatty acids was considered to be treated rather than untreated. The decision to initially code these individuals as untreated was based on evidence that omega-3 is not preventative of transition to psychosis^96^. In the third, only the 16 cohort studies were used in the model development and internal validation. This is because these studies provide the best quality evidence for prediction model building^97^. In the fourth, the following studies; Follow-up of the “At Risk Mental State” (FARMS), Minocyclin and/or Omega-3 Fatty Acids Added to Treatment as Usual for At Risk Mental States (NAYAB) and the Support for Wellness Achievement Program (SWAP) were removed from the data available for model building as they did not report any global functioning scores and thus all their values were imputed.

## Supplementary information

Supplementary Material

## Data Availability

No new data were created or analysed in this study. All original data sources described in this article are available on request from the relevant author(s).
