# Supplementary material for: Clinical prediction model for transition to psychosis in individuals meeting At Risk Mental State criteria
Source: Schizophrenia (Heidelb). 2025 Feb 27;11(1):29. doi: 10.1038/s41537-025-00582-5 (PMC11865318; doi:10.1038/s41537-025-00582-5)

Supplementary Material

# PRIMSA-style diagram

Supplementary Figure 1: PRISMA-style diagram depicting the flow of studies and individuals through the systematic review and meta-analysis
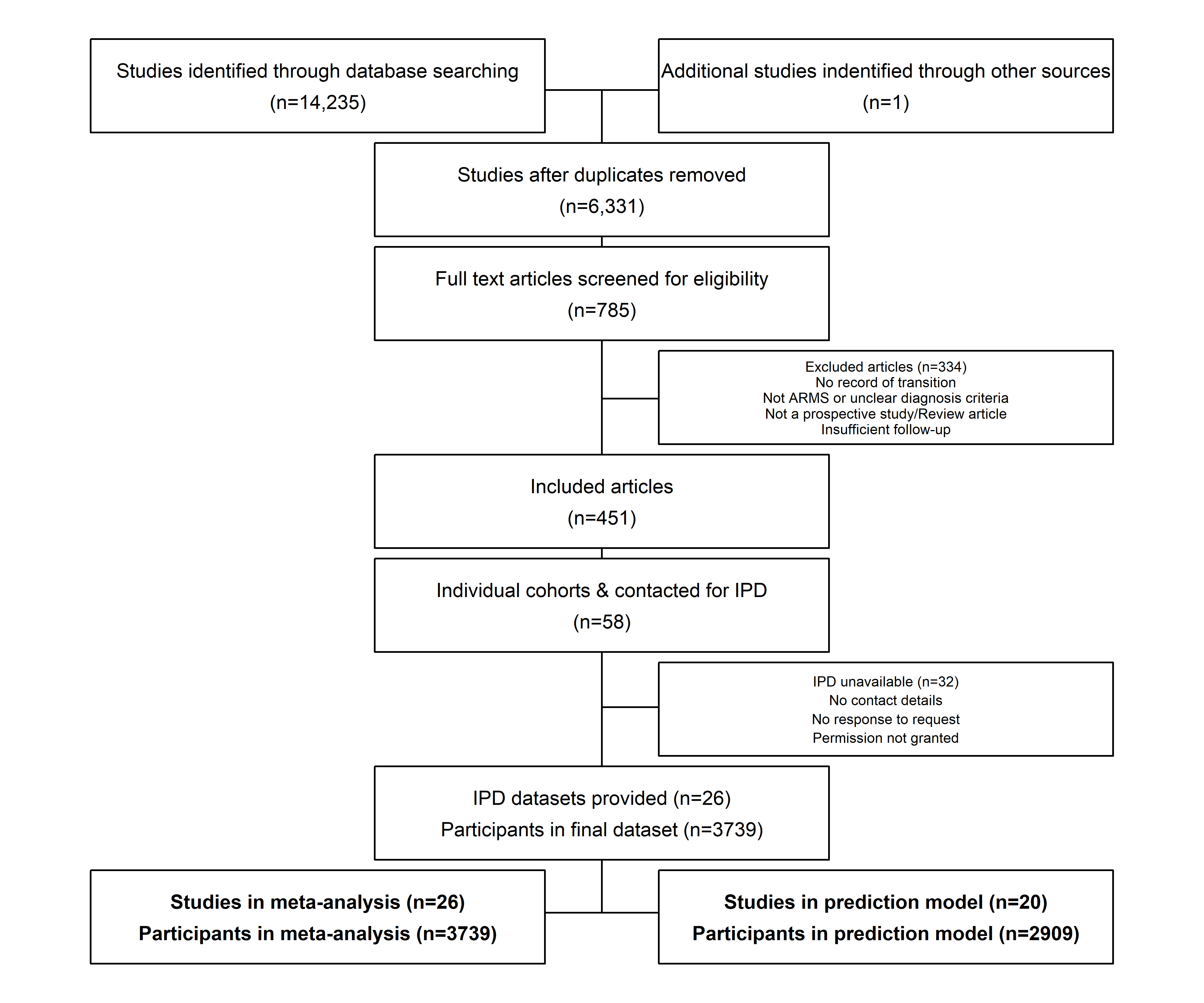


# Data Harmonisation for GAF and SOFAS

| **GAF** | **SOFAS** |
| --- | --- |
| 1 | ≤5.3 |
| 2 | 5.6 |
| 3 | 6.0 |
| 4 | 6.5 |
| 5 | 7.1 |
| 6 | 7.8 |
| 7 | 8.6 |
| 8 | 7.3 |
| 9 | 8.3 |
| 10 | 7.6 |
| 11 | 8.6 |
| 12 | 9.8 |
| 13 | 11.1 |
| 14 | 12.4 |
| 15 | 13.8 |
| 16 | 15.2 |
| 17 | 16.6 |
| 18 | 18.0 |
| 19 | 19.4 |
| 20 | 20.7 |
| 21 | 22.0 |
| 22 | 23.2 |
| 23 | 24.4 |
| 24 | 25.5 |
| 25 | 26.6 |
| 26 | 27.7 |
| 27 | 28.7 |
| 28 | 29.8 |
| 29 | 30.8 |
| 30 | 31.8 |
| 31 | 32.7 |
| 32 | 33.7 |
| 33 | 34.6 |
| 34 | 35.5 |
| 35 | 36.3 |
| 36 | 37.2 |
| 37 | 38.2 |
| 38 | 39.1 |
| 39 | 40.0 |
| 40 | 41.0 |
| 41 | 41.9 |
| 42 | 42.8 |
| 43 | 43.7 |
| 44 | 44.6 |
| 45 | 45.5 |
| 46 | 46.3 |
| 47 | 47.2 |
| 48 | 48.2 |
| 49 | 49.1 |
| 50 | 50.1 |
| 51 | 51.0 |
| 52 | 51.9 |
| 53 | 52.9 |
| 54 | 53.8 |
| 55 | 54.8 |
| 56 | 55.8 |
| 57 | 56.8 |
| 58 | 57.8 |
| 59 | 58.9 |
| 60 | 60.0 |
| 61 | 61.0 |
| 62 | 62.1 |
| 63 | 63.2 |
| 64 | 64.2 |
| 65 | 65.3 |
| 66 | 66.3 |
| 67 | 67.4 |
| 68 | 68.4 |
| 69 | 69.3 |
| 70 | 70.2 |
| 71 | 70.9 |
| 72 | 71.7 |
| 73 | 72.6 |
| 74 | 73.6 |
| 75 | 74.8 |
| 76 | 76.0 |
| 77 | 77.2 |
| 78 | 78.3 |
| 79 | 79.1 |
| 80 | 79.9 |
| 81 | 80.6 |
| 82 | 81.4 |
| 83 | 82.3 |
| 84 | 83.4 |
| 85 | 84.5 |
| 86 | 85.8 |
| 87 | 87.0 |
| 88 | 88.1 |
| 89 | 89.0 |
| 90 | 89.8 |
| 91 | 90.6 |
| 92 | 91.3 |
| 93 | 92.3 |
| 94 | 93.4 |
| 95 | 94.5 |
| 96 | 95.8 |
| 97 | 96.9 |
| 98 | 98.4 |
| 99 | 98.0 |
| 100 | ≥98.0 |

# IPD-MA – Forest Plots for Each Variable (Univariable Analyses)

## Age


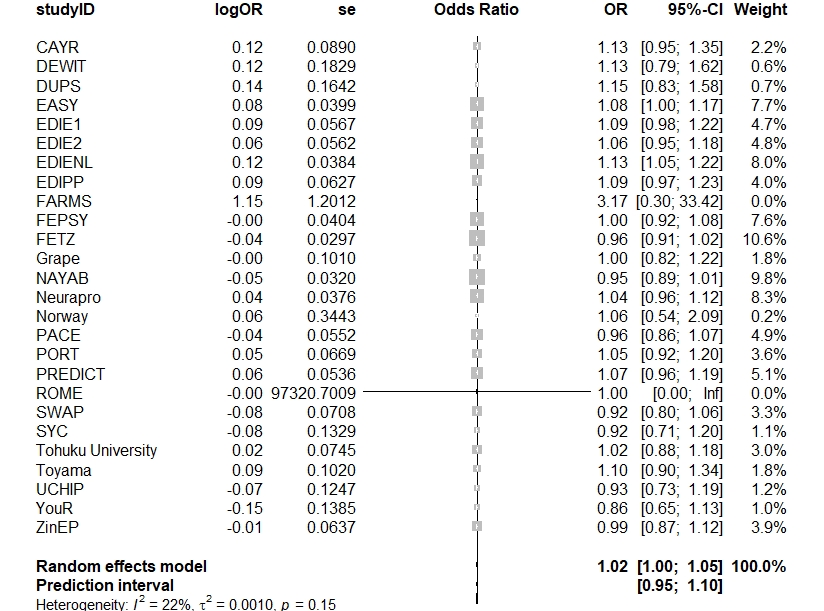


## Gender


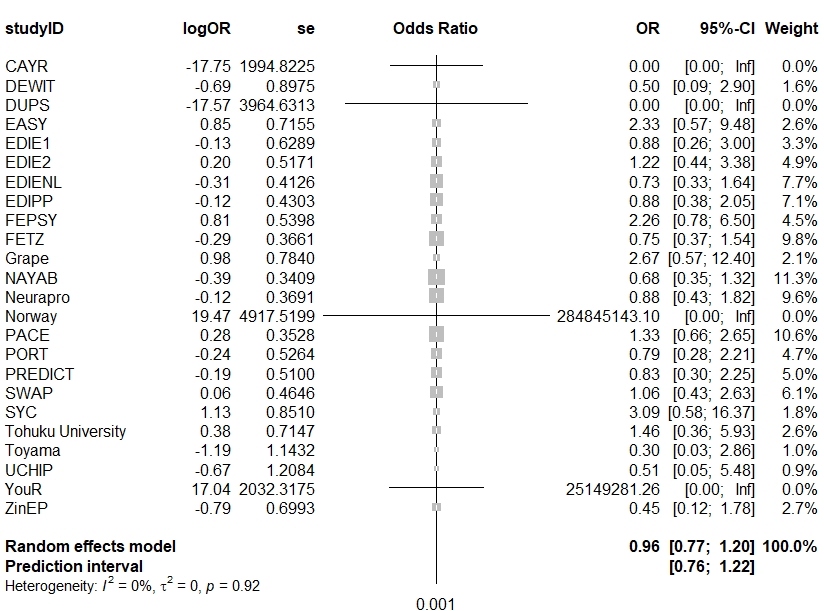


## Genetic Risk of Psychosis


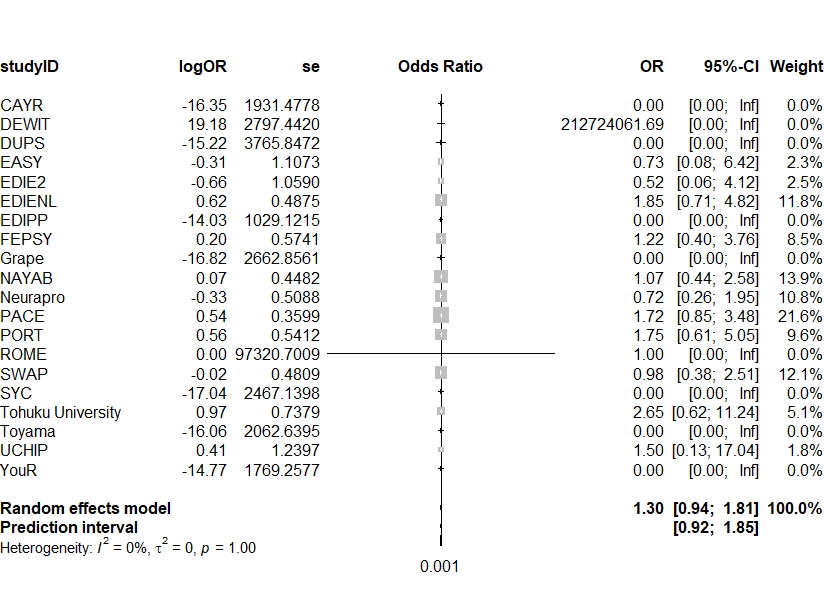


## Global Functioning Score


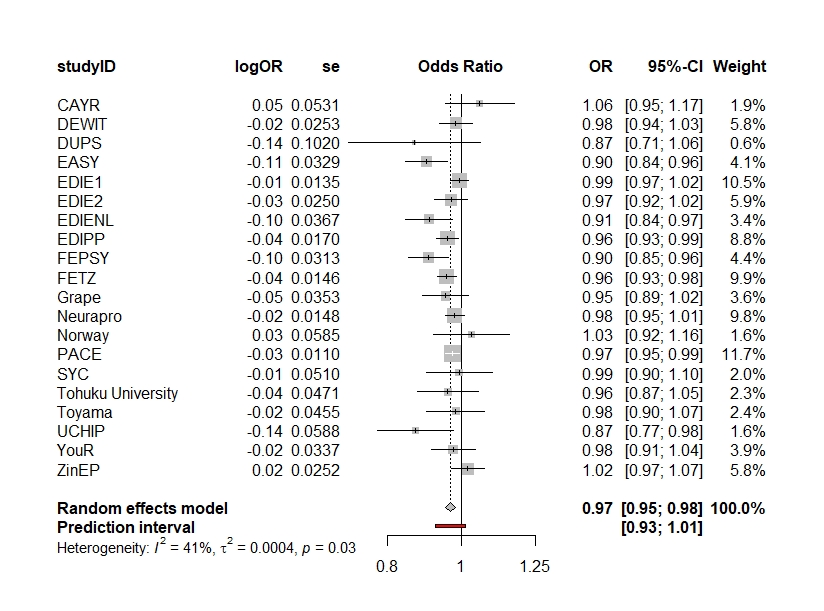


## Disorders of Thought Content


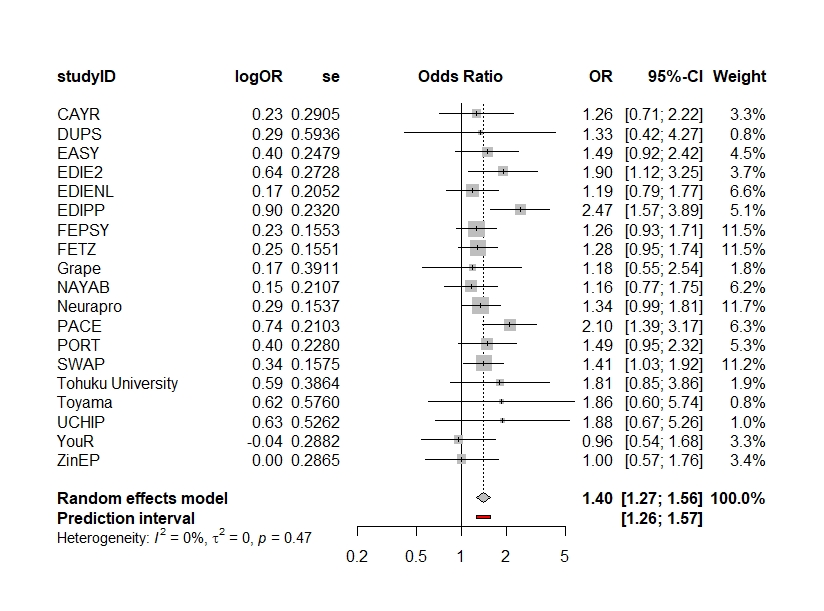


## Perceptual Abnormality


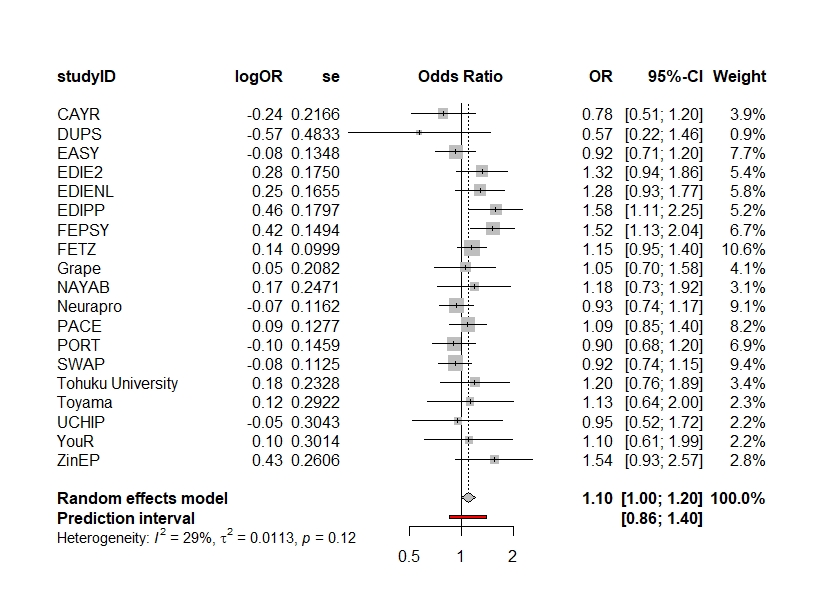


## Disorganised Speech


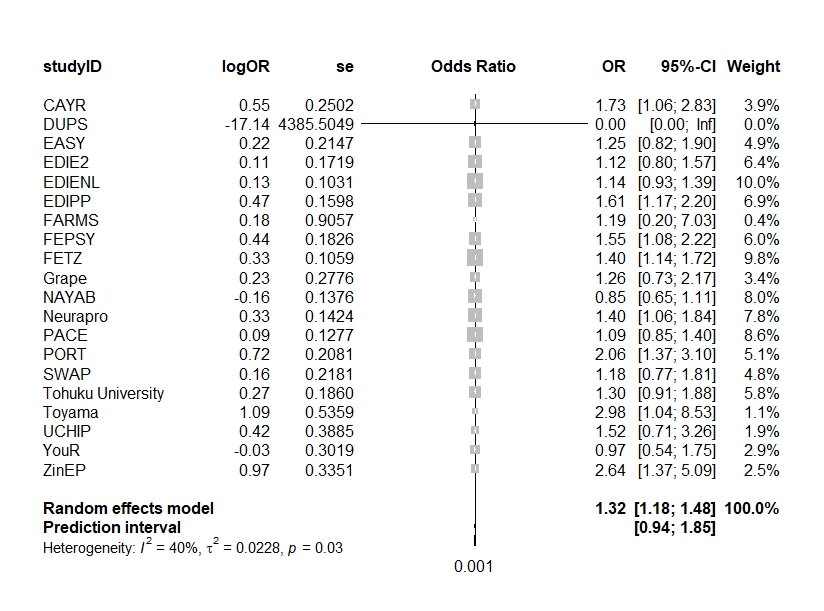


### Antidepressants


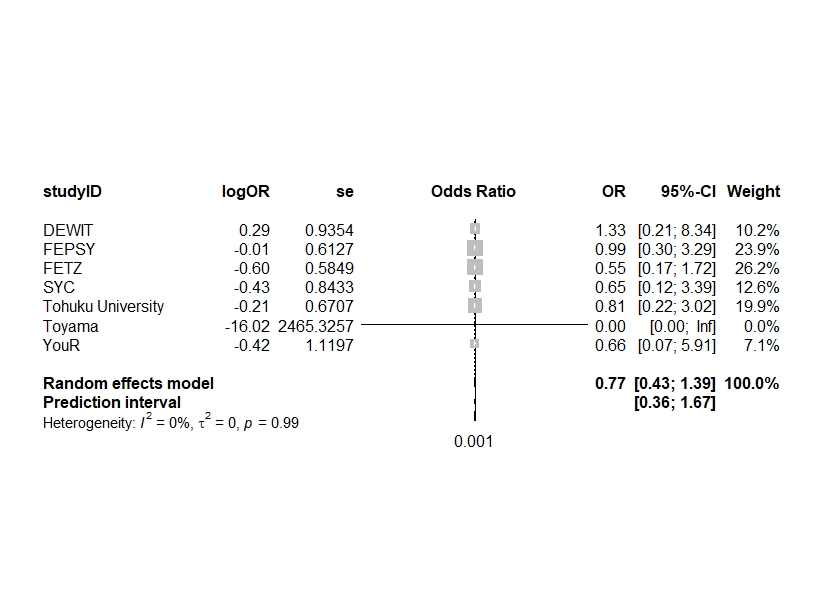


## Antipsychotic Medications


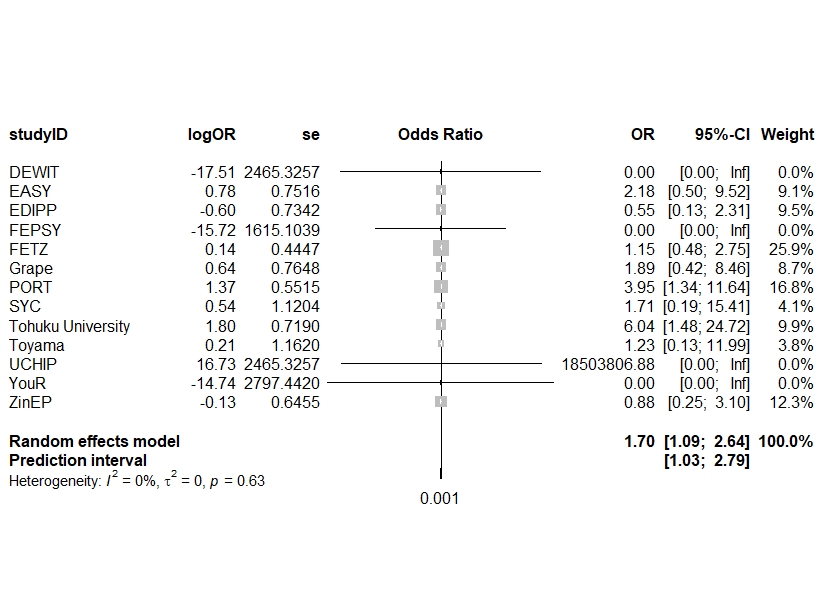


## Anxiety


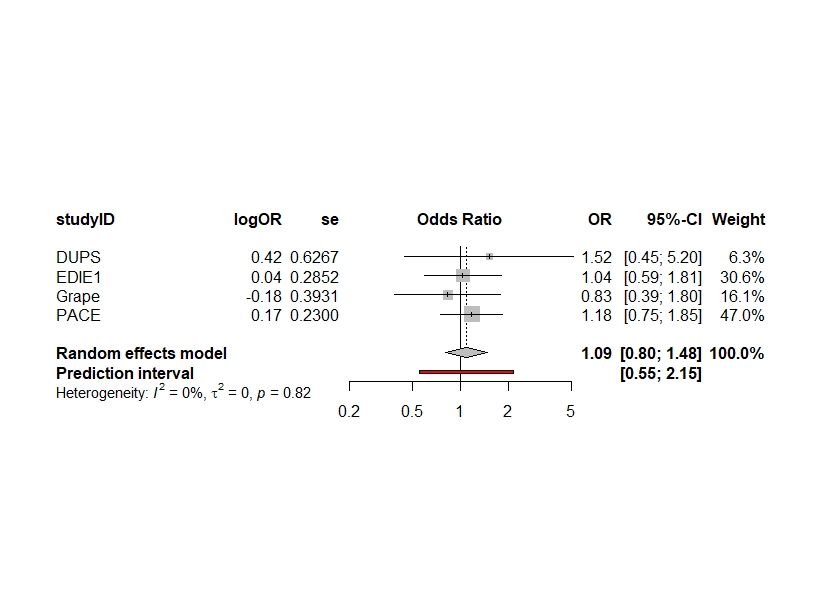


## CBT


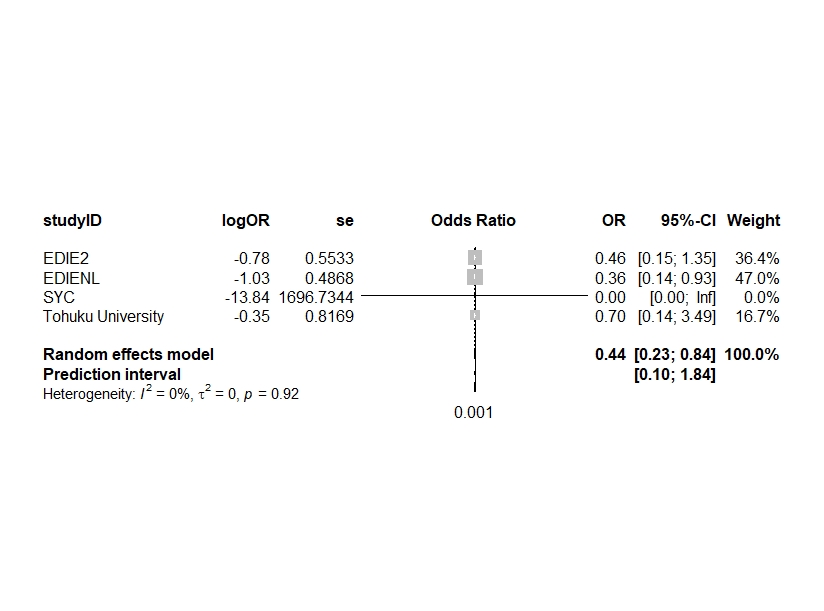


## Depression

### Binary


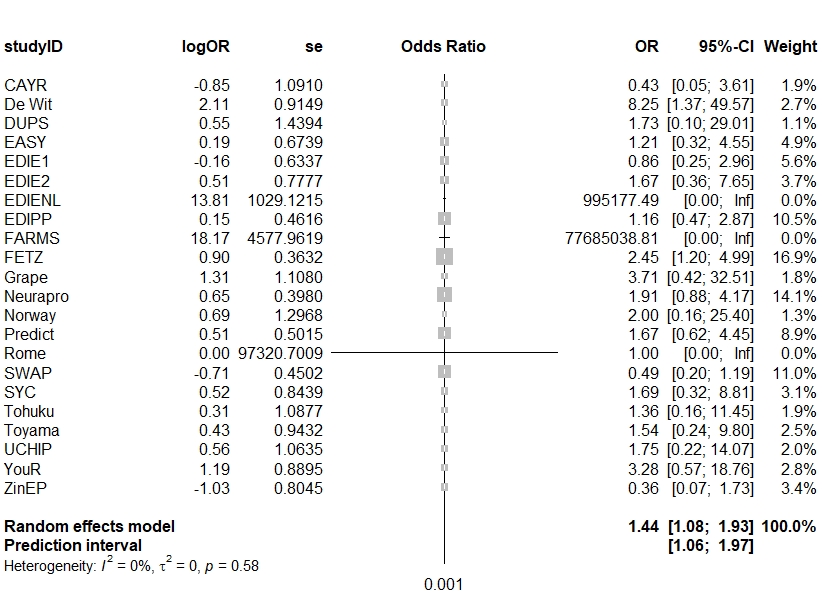


### Continuous


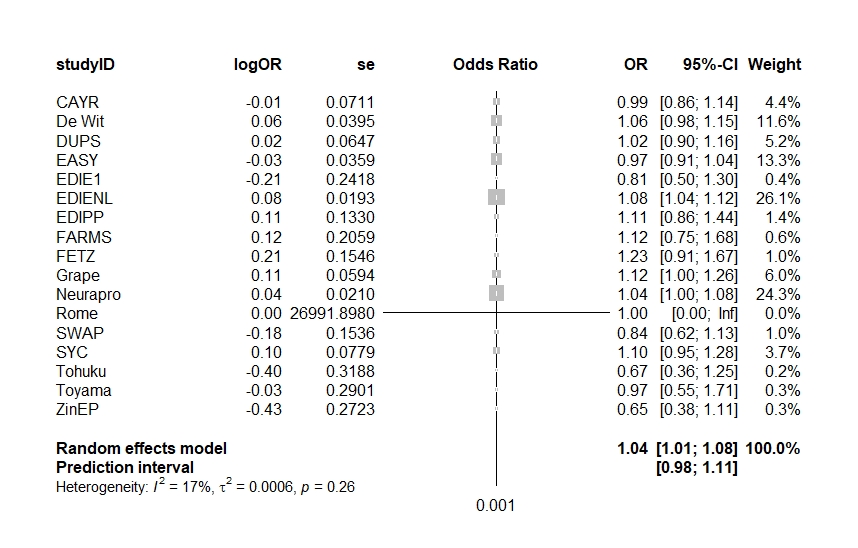


### Continuous with depression specific metrics only


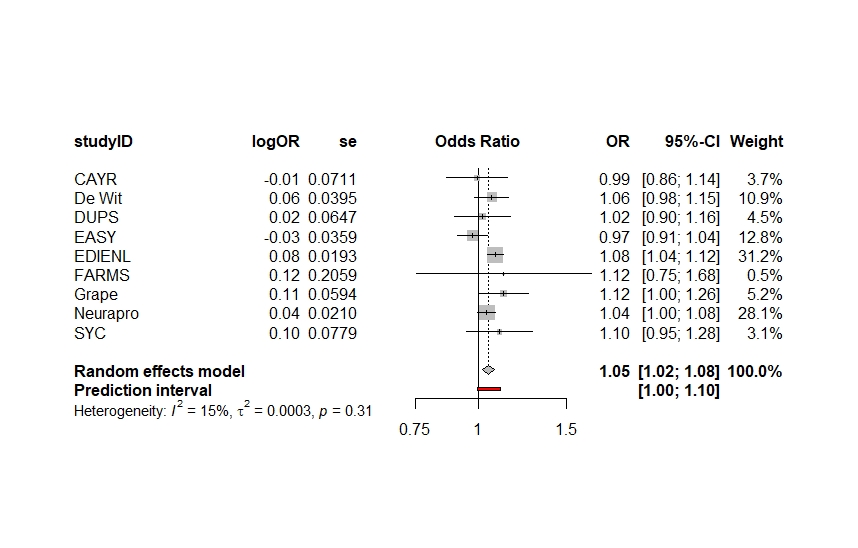


## Negative Symptoms

### PANSS


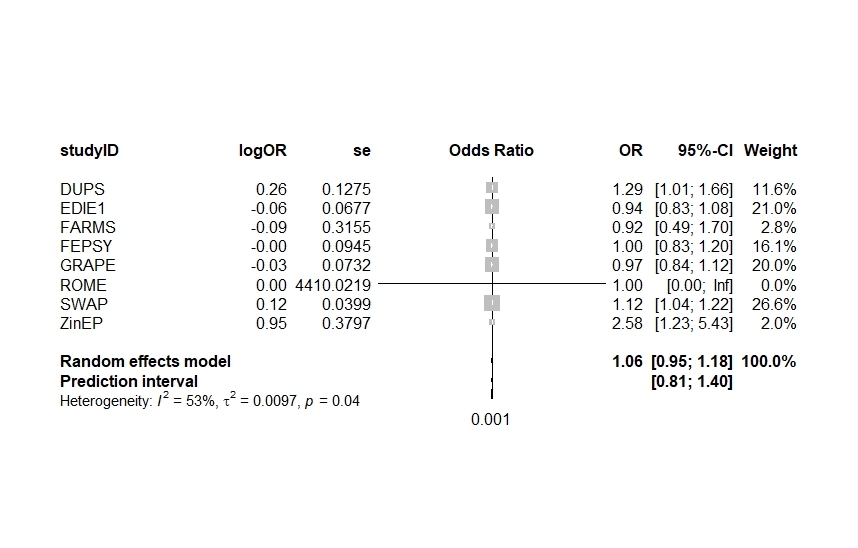


### SANS


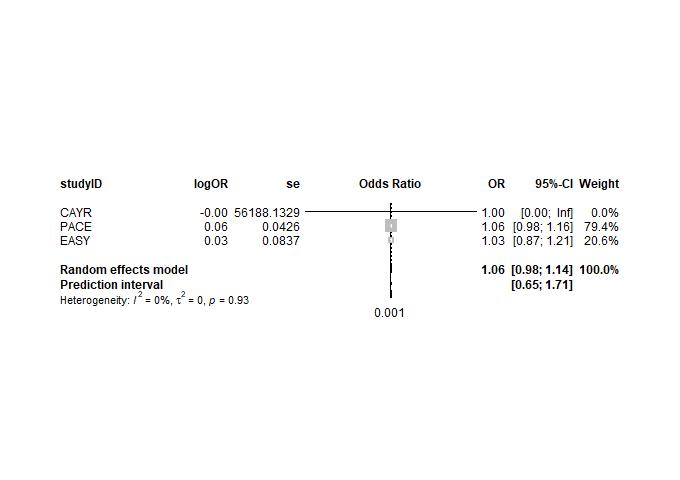


### SIPS


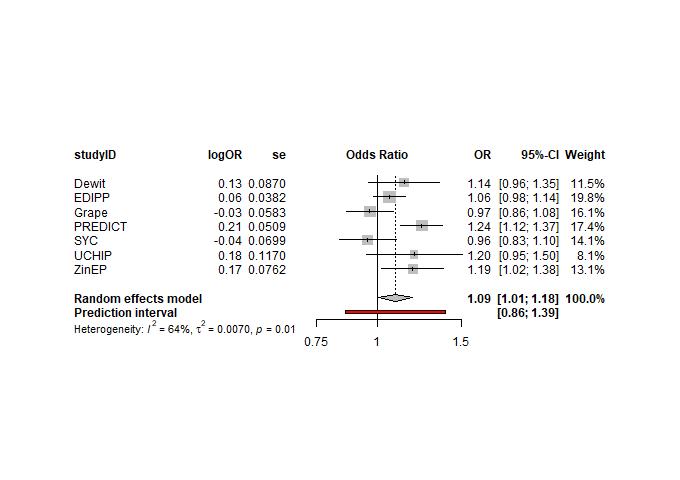


### Combined


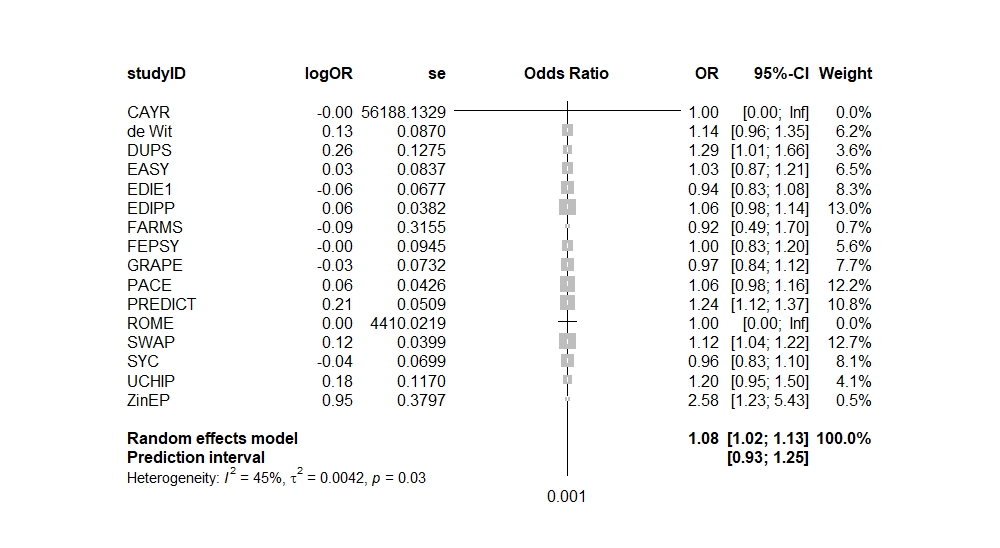


## Neurocognitive Variables

### Processing Speed


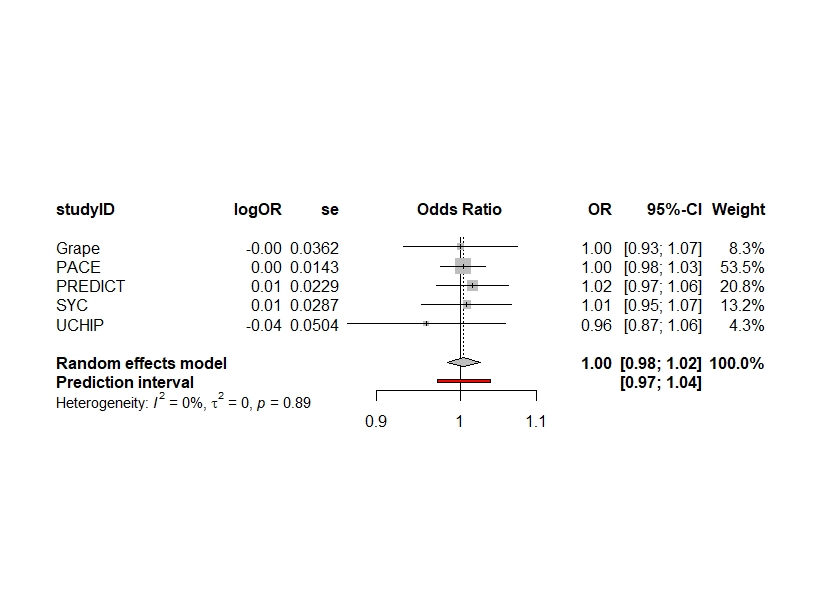


### Verbal Fluency


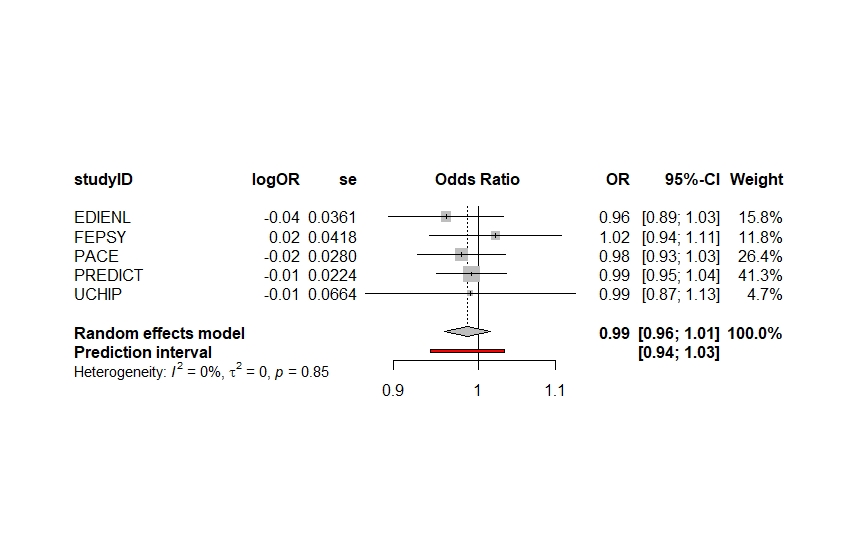


### Verbal Learning and Memory


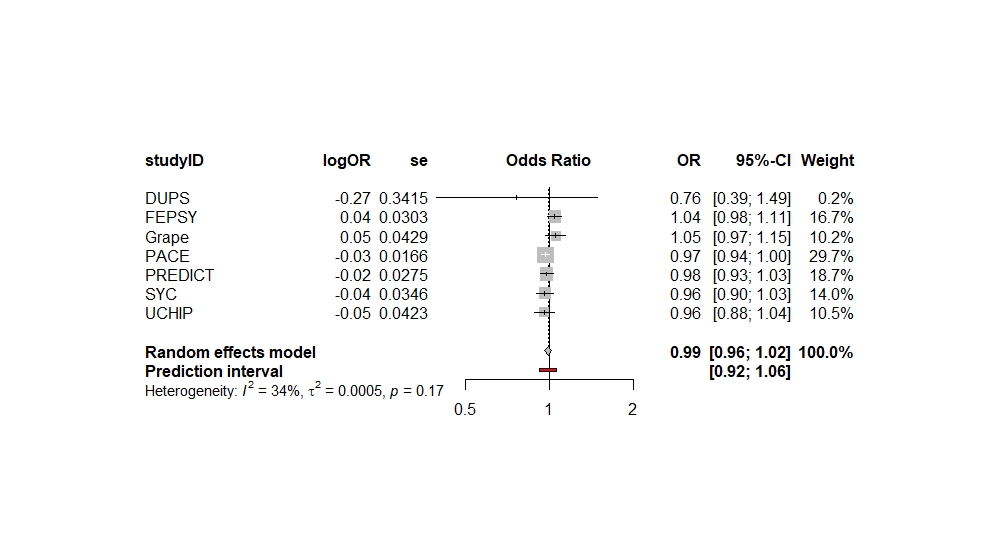


### Executive Function


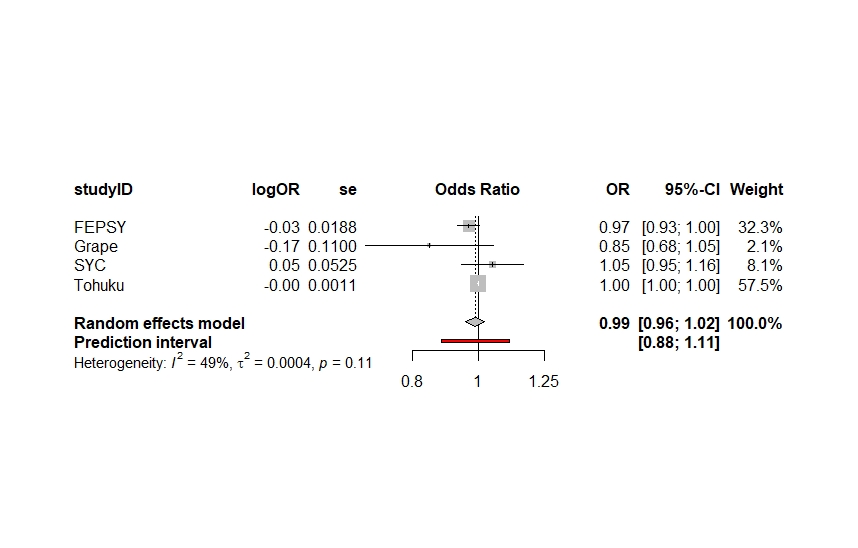


## Premorbid Adjustment

### Childhood (total)

####
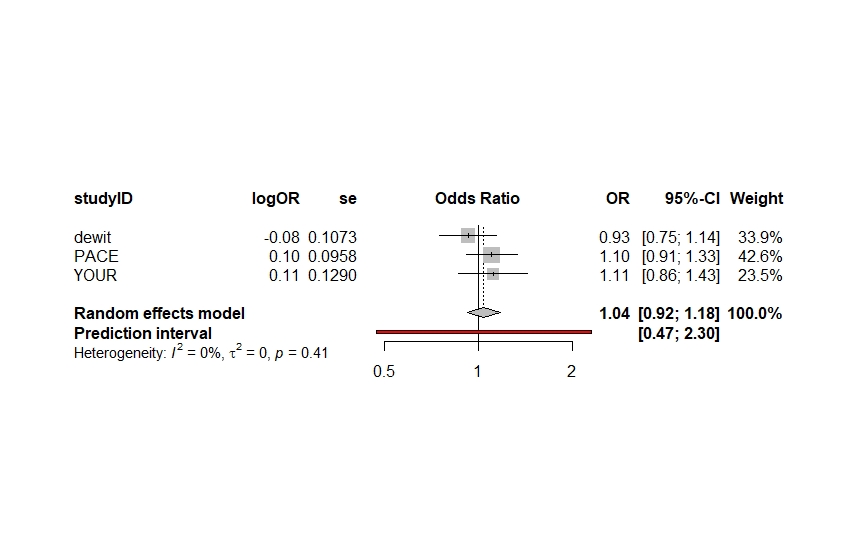


### Early Adolescence (total)


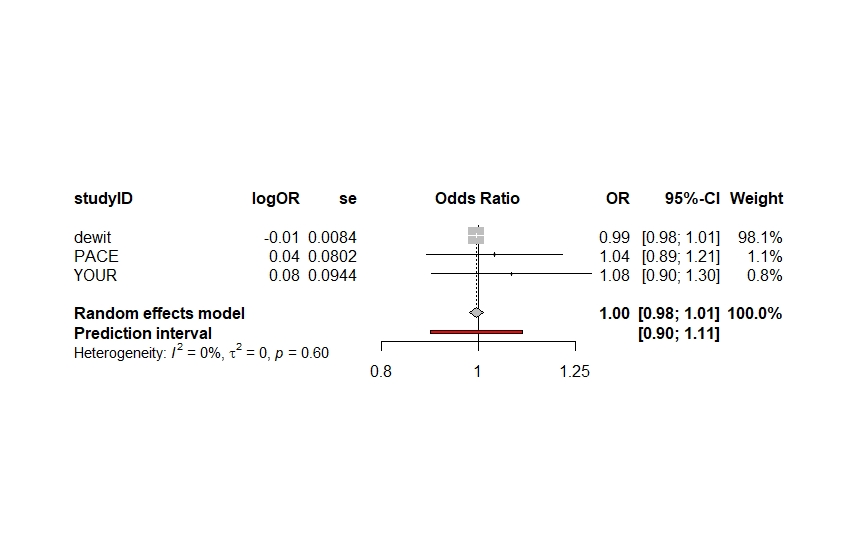


### Late Adolescence (total)


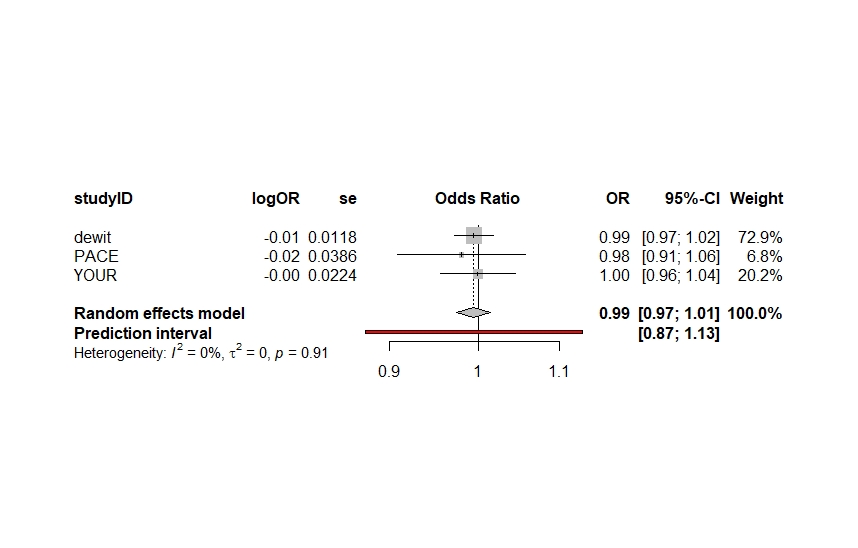


### Sub-scores

|  | **Odds Ratio [95% Confidence Interval]** | | |
| --- | --- | --- | --- |
| **Pre-morbid Adjustment Scale measures** | **Childhood** | **Early Adolescence** | **Late Adolescence** |
|  | **(Age 6-11)** | **(Age 12-15)** | **(Age 16-18)** |
| Sociability | 1.00 (0.95, 1.04) | 1.03 (0.89, 1.22) | 0.99 (0.96, 1.03) |
| Peer Relationship | 0.78 (0.46, 1.31) | 0.99 (0.95, 1.04) | 0.81 (0.59, 1.13) |
| Scholastic Performance | 0.90 (0.64, 1.27) | 0.99 (0.97, 1.02) | 1.00 (0.91, 1.10) |
| Adaptation to School | 0.90 (0.52, 1.58) | 0.99 (0.94, 1.05) | 1.00 (0.85, 1.17) |
| Sexual Relations | N/A | 0.99 (0.96, 1.03) | 0.95 (0.58, 1.54) |

## Substance Abuse

### Substance Abuse


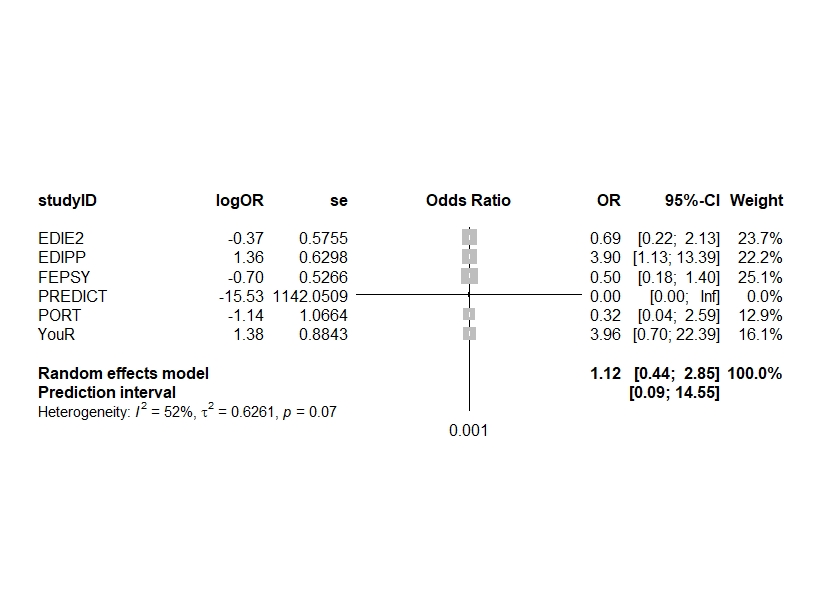


### Cannabis


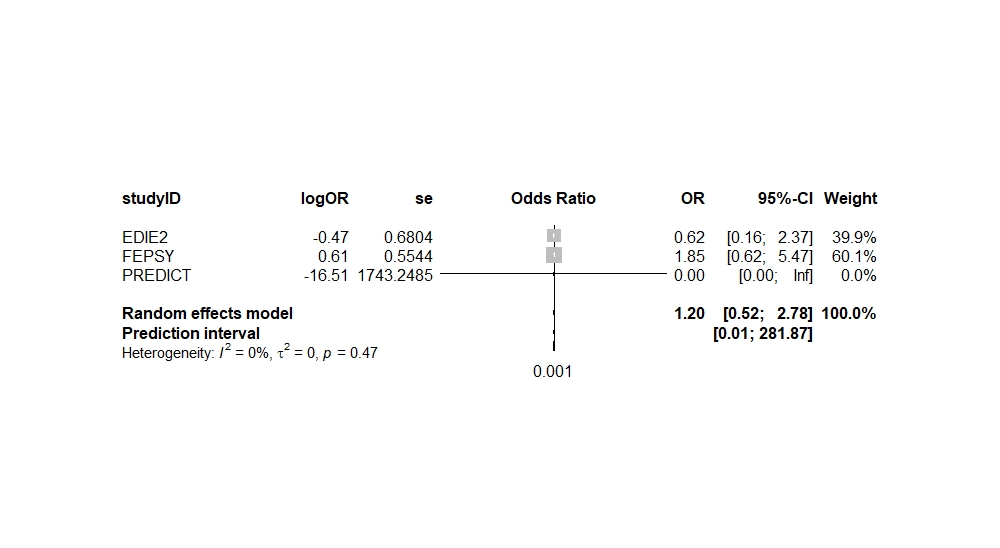


### Alcohol


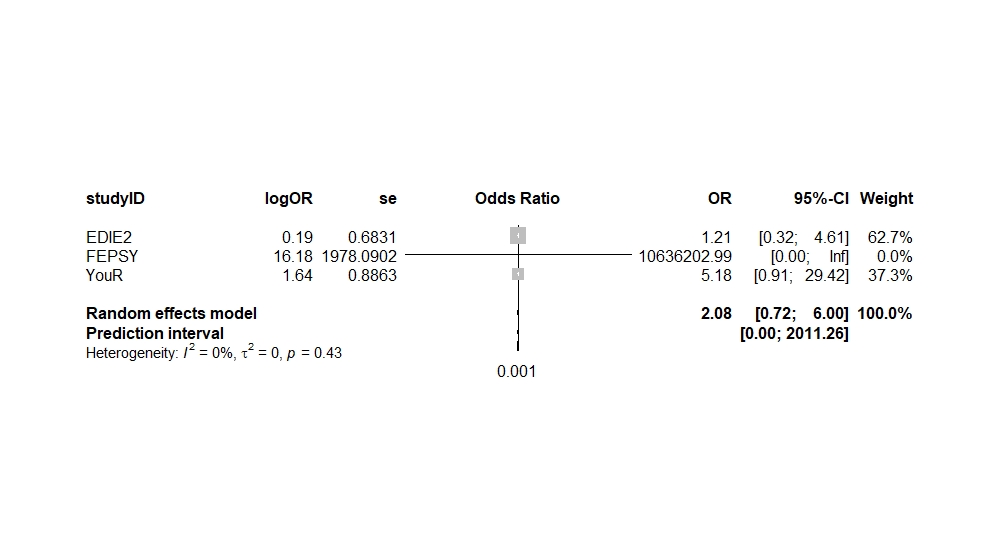


## Trauma

### Binary Variable

#### Physical Neglect


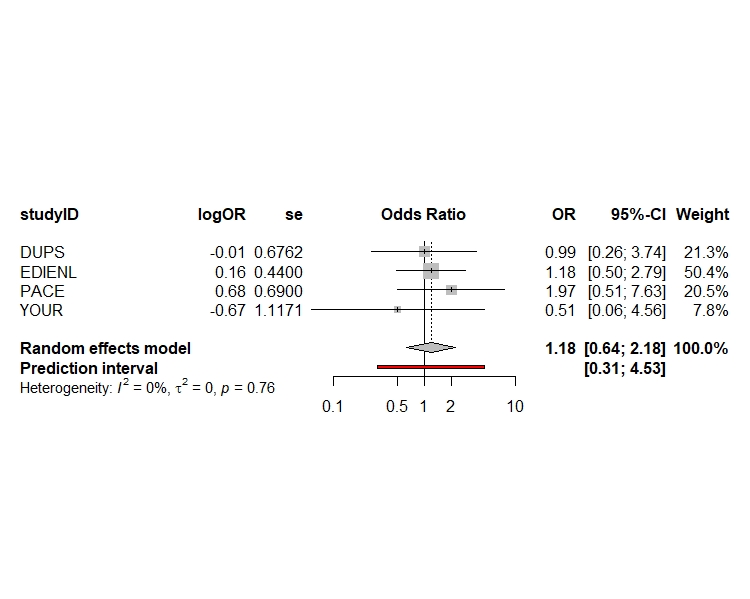


#### Emotional Neglect


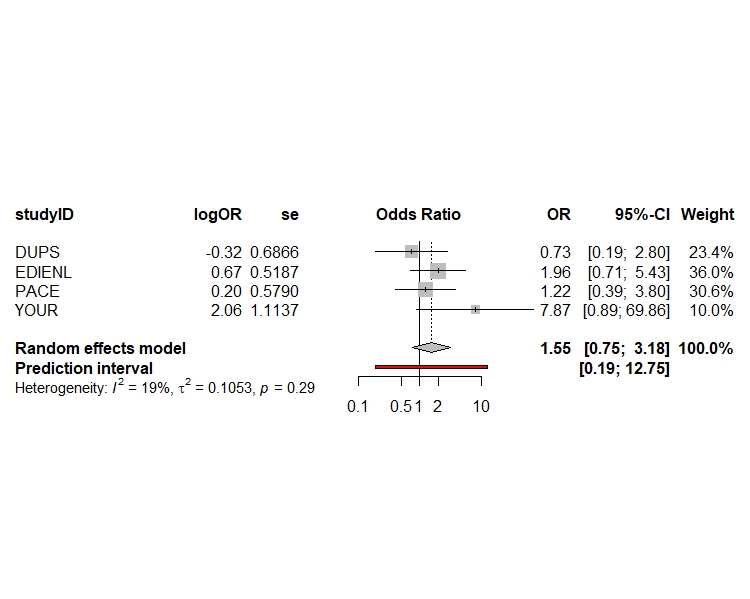


#### Physical Abuse


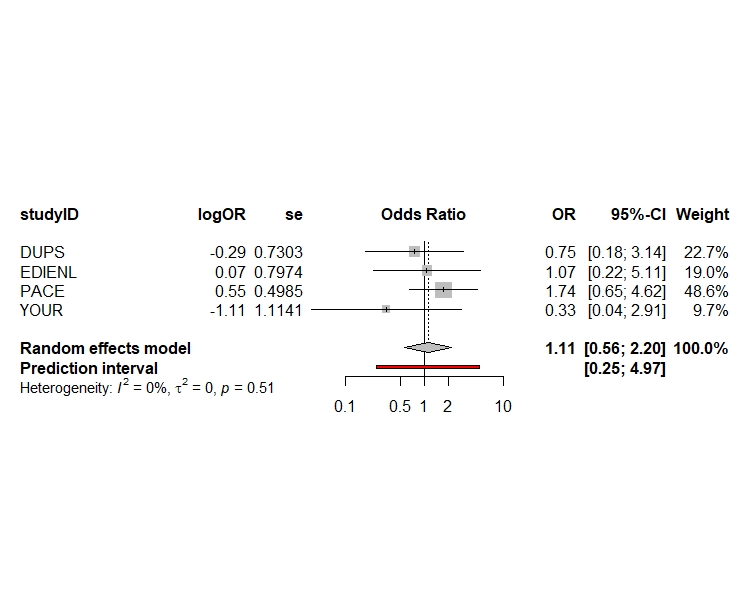


#### Emotional Abuse


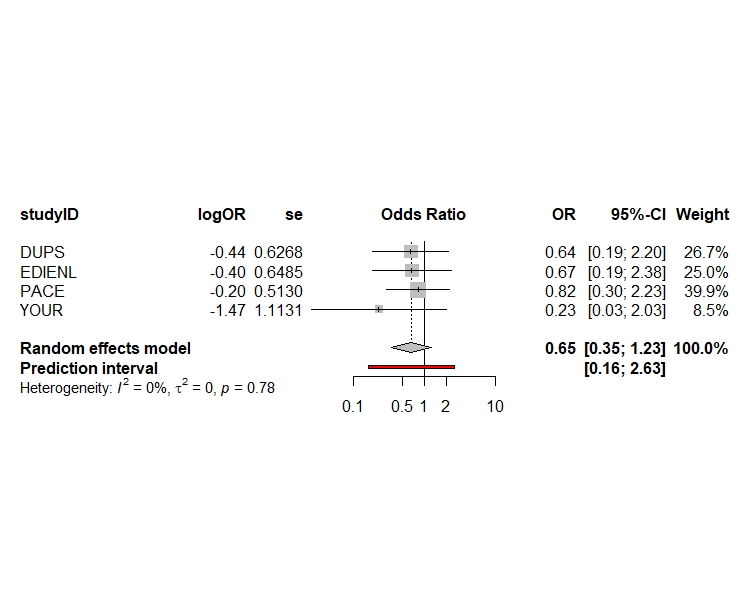


#### Sexual Abuse


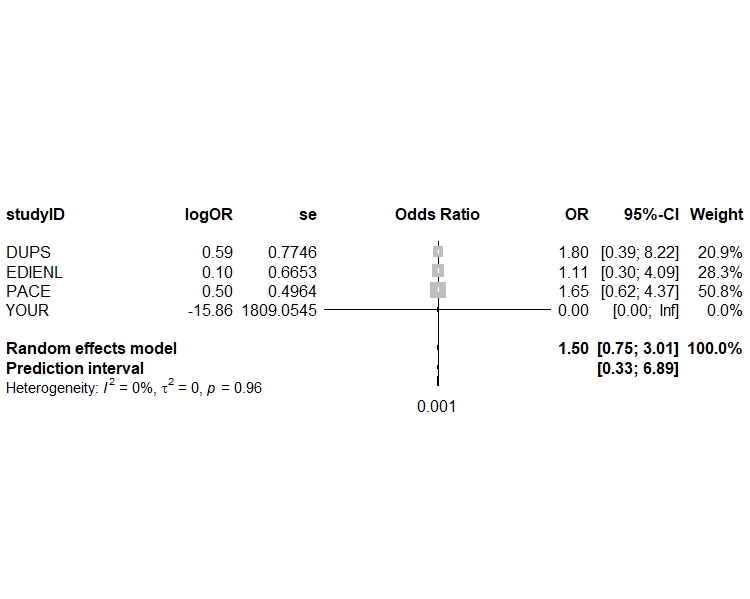


### Continuous Variable

#### Physical Neglect


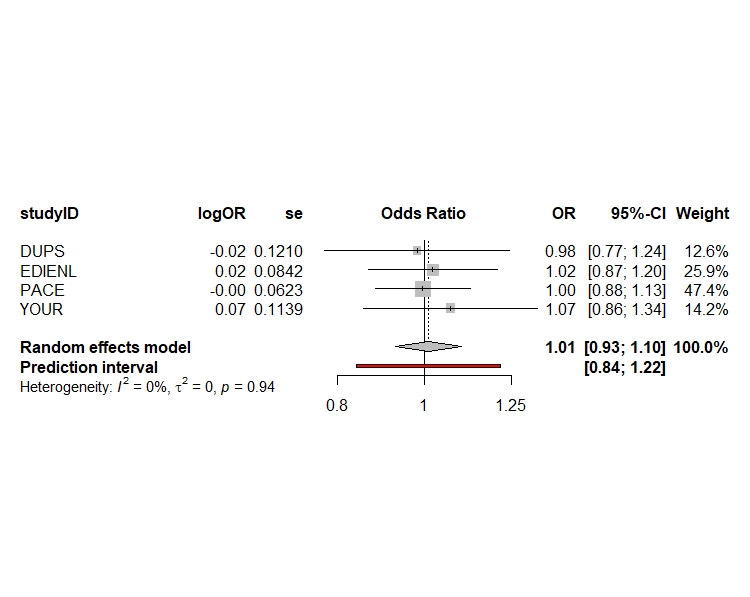
Emotional Neglect


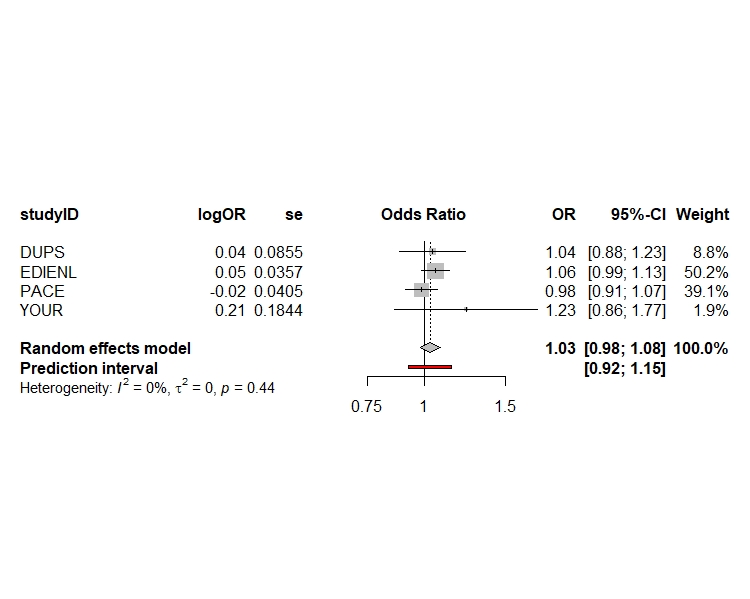


#### Physical Abuse


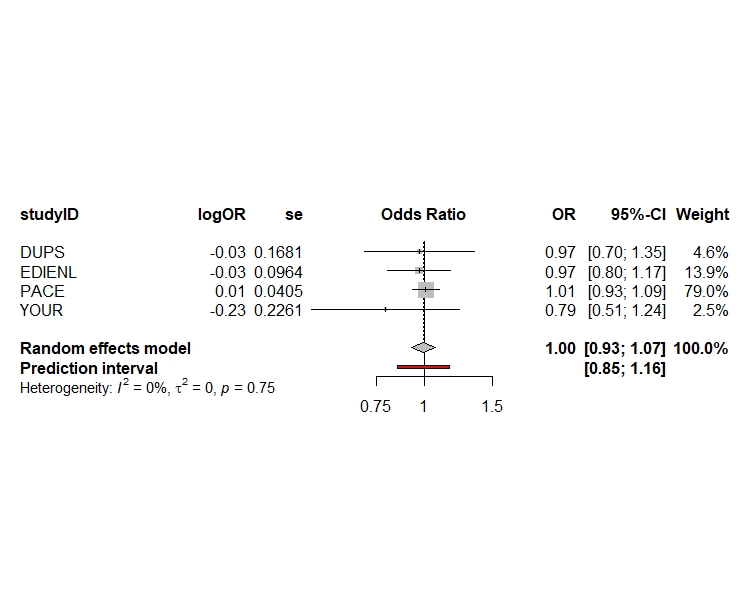


#### Emotional Abuse


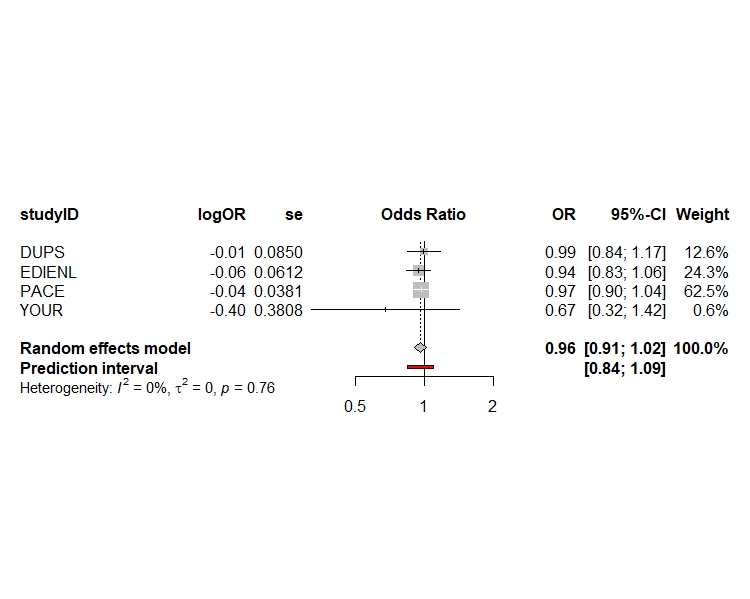


#### Sexual Abuse


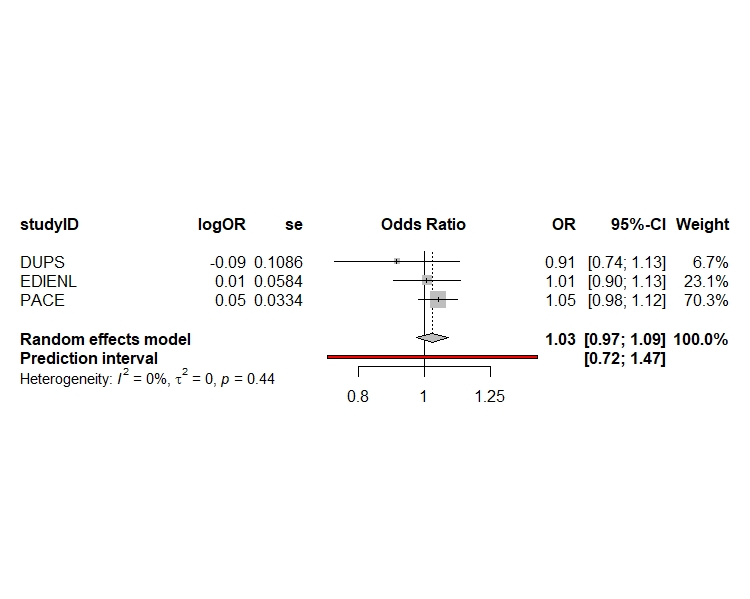


# Demographic Data

Supplementary Table 1: Demographic summary of people in each individual study within the IPD dataset– numbers are median (interquartile range) [range] unless otherwise stated. Missing values are in italics.

|  | **CAYR** | **DUPS** | **EASY** | **EDIE 2** | **EDIE-NL** | **EDIPP** | **FARMS** | **FEPSY** | **FETZ** | **Grape** | **NAYAB** | **Neurapro** | **PACE** | **PORT** | **SAFE** | **SWAP** | **Toyama Uni.** | **UCHIP** | **YouR** | **ZinEP** |
| --- | --- | --- | --- | --- | --- | --- | --- | --- | --- | --- | --- | --- | --- | --- | --- | --- | --- | --- | --- | --- |
| **Variable** | **(n=213)** | **(n=62)** | **(n=86)** | **(n=193)** | **(n=192)** | **(n=155)** | **(n=27)** | **(n=131)** | **(n=164)** | **(n=60)** | **(n=297)** | **(n=304)** | **(n=268)** | **(n=115)** | **(n=132)** | **(n=184)** | **(n=76)** | **(n=37)** | **(n=109)** | **(n=104)** |
| Gender, n (%) |  |  |  |  |  |  |  |  |  |  |  |  |  |  |  |  |  |  |  |  |
| Male | 124(58) | 42 (68) | 38 (44) | 122 (63) | 97 (51) | 88 (57) | 27 (100) | 97 (74) | 104 (63) | 35 (58) | 175 (59) | 139 (46) | 116 (43) | 55 (48) | 50 (38) | 124 (67) | 43 (57) | 23 (62) | 28 (26) | 62 (60) |
| Female | 79 (37) | 20 (32) | 48 (56) | 71 (37) | 95 (49) | 67 (49) | 0 (0) | 34 (26) | 60 (37) | 25 (42) | 122 (41) | 165 (54) | 152 (57) | 60 (52) | 82 (62) | 60 (33) | 33 (43) | 14 (38) | 81 (74) | 42 (40) |
| Missing | *10 (5)* |  |  |  |  |  |  |  |  |  |  |  |  |  |  |  |  |  |  |  |
| Genetic Risk, n (%) |  |  |  |  |  |  |  |  |  |  |  |  |  |  |  |  |  |  |  |  |
| No | 182 (85) | 59 (95) | 76 (88) | 173 (90) | 160 (83) | 153 (99) | 27 (100) | 97 (74) | 164(100) | 54 (90) | 253 (85) | 245 (81) | 194 (72) | 80 (70) | 112 (85) | 184(100) | 66 (87) | 30 (81) | 104 (95) | 104(100) |
| Yes | 31 (15) | 3 (5) | 10 (12) | 20 (10) | 32 (17) | 2 (1) | 0 (0) | 34 (26) | 0 (0) | 6 (10) | 44 (15) | 59 (19) | 74 (28) | 35 (30) | 20 (15) | 0 (0) | 10 (13) | 7 (19) | 5 (5) | 0 (0) |
| Age (years) | 19  (16, 22)  [13, 33]  *12* | 19  (16, 22)  [12, 29] | 18  (16, 24)  [13, 43] | 19  (18, 23) [14, 34] | 22  (19, 27) [13, 36] | 16  (14, 19) [12, 26]  *14* | 16  (15, 17) [12, 18] | 22  (21, 28) [18, 51] | 24  (20, 30) [15, 40] | 20  (17, 21) [14, 32] | 24  (20, 29) [16, 35] | 18  (16, 22) [13, 39] | 18  (17, 21) [14, 29] | 18  (16, 21) [15, 32] | 18  (17, 22) [14, 35] | 20  (19, 22) [15, 31] | 17  (16, 19) [13, 31] | 17  (16, 21) [12, 29] | 20  (18, 24) [16, 34] | 17  (15, 22) [13, 35] |
| Combined Score: Disorganised thought content | 3  (3, 5)  [0, 6]  *3* | 4  (3, 5)  [1, 6] | 5  (3, 5)  [0, 6] | 4  (3, 5)  [0, 6]  *1* | 5  (4, 5)  [0, 6] | 4  (3, 5)  [1, 6] | 4  (3, 4)  [2, 5] | 0  (0, 0)  [0, 6] | 4  (3, 4)  [0, 6] | 4  (3, 5)  [0, 5]  *5* | 4  (4, 5)  [2, 5] | 4  (3, 5)  [0, 6] | 2  (1, 3)  [0, 4]  *10* | 4  (3, 4)  [0, 6] | 5  (4, 5)  [0, 6] | 4  (2, 5)  [0, 6] | 4  (3, 5)  [0, 5] | 4  (3, 5)  [1, 6] | 4  (3, 5)  [0, 6] | 3  (3, 4)  [1, 6]  *19* |
| Combined Score: Perceptual abnormalities | 3  (2, 4)  [0, 6]  *2* | 3  (1, 4)  [0, 5] | 4  (0, 5)  [0, 6] | 4  (2, 4)  [0, 6] | 4  (3, 5)  [0, 6] | 4  (3, 4)  [0, 6] | 4  (4, 5)  [0, 5] | 2  (0, 3)  [0, 6] | 2  (0, 3)  [0, 6] | 2  (0, 4)  [0, 6]  *6* | 4  (4, 4)  [0, 4] | 4  (3, 4)  [0, 6] | 3  (2, 3)  [0, 4]  *10* | 0  (0, 4)  [0, 5] | 4  (3, 5)  [0, 6] | 3  (0, 4)  [0, 6] | 3  (2, 4)  [0, 6] | 3  (2, 4)  [0, 5] | 3  (3, 4)  [0, 6] | 3  (2, 4)  [0, 6]  *20* |
| Combined Score: Disorganised speech | 2  (0, 3)  [0, 6]  *5* | 0  (0, 2)  [0, 6] | 0  (0, 0)  [0, 5] | 2  (0, 3)  [0, 5] | 3  (0, 5)  [0, 6] | 2  (1, 3)  [0, 6] | 2  (1, 3)  [0, 4] | 0  (0, 3)  [0, 4] | 2  (0, 3)  [0, 6] | 0  (0, 2)  [0, 4]  *5* | 3  (2, 4)  [0, 5] | 2  (0, 2)  [0, 5] | 3  (2, 3)  [0, 4]  *10* | 2  (0, 3)  [0, 5] | 5  (2, 6)  [0, 6] | 0  (0, 0)  [0, 5] | 3  (2, 3)  [0, 5]  *1* | 2  (1, 3)  [0, 5] | 2  (0, 3)  [0, 4] | 2  (1, 2)  [0, 5]  *19* |
| Global Functioning Scale, mean (standard deviation) [range] | 53 (12) [15, 85]  *62* | 51 (11) [29, 75] | 52 (14)  [21, 88] | 50 (11) [21, 87] | 47 (5) [30, 57] | 40 (13) [10, 70]  *1* | N/A  *27* | 55 (11) [30, 90] | 49 (15) [25, 91] | 49 (12) [21, 73]  *8* | N/A  *297* | 54 (13) [1, 86]  *6* | 65 (16) [25, 96] | 50 (8) [35, 71] | 48 (7) [30, 65] | N/A  *184* | 49 (10) [28, 68] | 52 (12)  [25, 76] | 55 (12) [21, 91] | 52 (13)  [20, 80]  *1* |
| Treated, n (%) |  |  |  |  |  |  |  |  |  |  |  |  |  |  |  |  |  |  |  |  |
| No | 0 (0) | 0 (0) | 0 (0) | 75 (39) | 0 (0) | 0 (0) | 0 (0) | 0 (0) | 0 (0) | 0 (0) | 0 (0) | 0 (0) | 0 (0) | 9 (8) | 37 (28) | 0 (0) | 0 (0) | 2 (5) | 37 (34) | 0 (0) |
| Yes | 0 (0) | 0 (0) | 14 (16) | 118 (61) | 77 (40) | 20 (13) | 0 (0) | 33 (25) | 46 (28) | 22 (37) | 0 (0) | 0 (0) | 0 (0) | 106 (92) | 95 (72) | 0 (0) | 20 (26) | 34 (92) | 36 (33) | 44 (42) |
| Not known | 213 (100) | 62 (100) | 72 (84) | 0 (0) | 115 (60) | 135 (87) | 27 (100) | 98 (75) | 118 (72) | 38 (63) | 297 (100) | 304 (100) | 268 (100) | 0 (0) | 0 (0) | 184 (100) | 56 (74) | 1 (3) | 36 (33) | 60 (58) |
| Transitioned to psychosis, n (%) |  |  |  |  |  |  |  |  |  |  |  |  |  |  |  |  |  |  |  |  |
| No | 206 (97) | 60 (97) | 75 (87) | 176 (91) | 164 (85) | 128 (83) | 26 (96) | 114 (87) | 117 (71) | 52 (87) | 252 (85) | 271 (89) | 228 (85) | 98 (85) | 122 (92) | 160 (87) | 71 (93) | 35 (95) | 103 (94) | 92 (88) |
| Yes | 7 (3) | 2 (3) | 11 (13) | 17 (9) | 28 (15) | 27 (17) | 1 (4) | 17 (13) | 47 (29) | 8 (13) | 45 (15) | 33 (11) | 40 (15) | 17 (15) | 10 (8) | 24 (13) | 5 (7) | 2 (5) | 6 (6) | 12 (12) |

# Model fitting prior to shrinkage

## Model estimates prior to shrinkage for model optimism

Supplementary Table 2: Parsimonious multivariable model for risk of transition to psychosis according to the IPD dataset (not adjusted for shrinkage)

|  | **Odds Ratio** |
| --- | --- |
| **Variable** | **(95% CI)** |
| *Intercept, log odds ratio (standard error)* | *-3.45 (0.67)* |
| Combined score: Disorganised thought content (linear) | 1.35 (1.21, 1.50) |
| Combined score: Disorganised speech (linear) | 1.23 (1.14, 1.33) |
| Global Functioning Scale (linear) | 0.97 (0.96, 0.98) |
| Treatment |  |
| No | 1.00 |
| Yes | 0.81 (0.40, 1.65) |
| Not known | 0.97 (0.44, 2.16) |

## Model performance measures prior to shrinkage for model optimism

- C statistic: 0.72 (0.69, 0.74)
- E/0: 1

Supplementary Figure 2: Calibration plot comparing observed risk of transition to psychosis with that observed in the IPD dataset


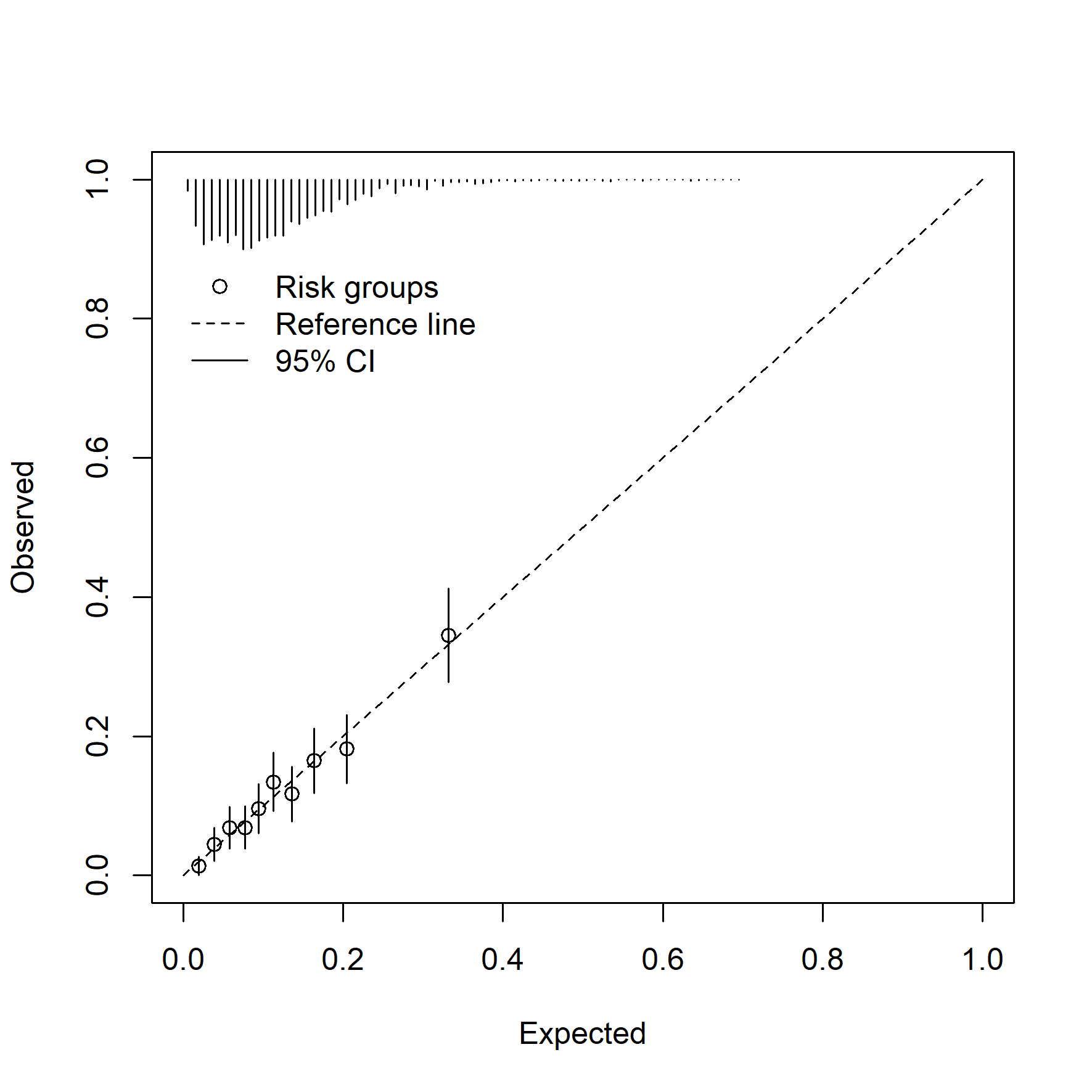


# Sensitivity Analyses

## Sensitivity Analysis: APS instead of genetic risk

Supplementary Table 3: APS characteristics of individuals within the IPD dataset

|  | **Did not transition** | **Transitioned** | **Total** |
| --- | --- | --- | --- |
| **Variable** | **(n=2550)** | **(n=359)** | **(n=2909)** |
| APS, n (%) |  |  |  |
| No | 291 (11) | 38 (11) | 329 (11) |
| Yes | 2259 (89) | 321 (89) | 2580 (89) |

## Sensitivity Analysis: Omega-3 fatty acids as treatment

Supplementary Table 4: Treatment within the IPD dataset when omega-3 fatty acids are considered as treatment

|  | **Did not transition** | **Transitioned** | **Total** |
| --- | --- | --- | --- |
| **Variable** | **(n=2550)** | **(n=359)** | **(n=2909)** |
| Treated (including omega-3), n (%) |  |  |  |
| No | 147 (6) | 13 (4) | 160 (6) |
| Yes | 728 (29) | 90 (25) | 818 (28) |
| Not known | 1675 (66) | 256 (71) | 1931 (66) |

Supplementary Table 5: Parsimonious multivariable model for risk of transition to psychosis according to the IPD dataset with omega-3 fatty acids considered as treatment (not adjusted for shrinkage)

|  | **Odds Ratio** |
| --- | --- |
| **Variable** | **(95% CI)** |
| *Intercept, log odds ratio (standard error)* | *-3.50 (0.67)* |
| Combined score: Disorganised thought content (linear) | 1.35 (1.22, 1.50) |
| Combined score: Disorganised speech (linear) | 1.23 (1.14, 1.33) |
| Global Functioning Scale (linear) | 0.97 (0.96, 0.98) |
| Treatment |  |
| No | 1.00 |
| Yes | 0.81 (0.40, 1.64) |
| Not known | 1.02 (0.47, 2.22) |

- C statistic: 0.72 (0.69, 0.74)
- E/0: 1

Supplementary Figure 3: Calibration plot comparing observed risk of transition to psychosis with that observed in the IPD dataset (people taking omega-3 coded as treated)


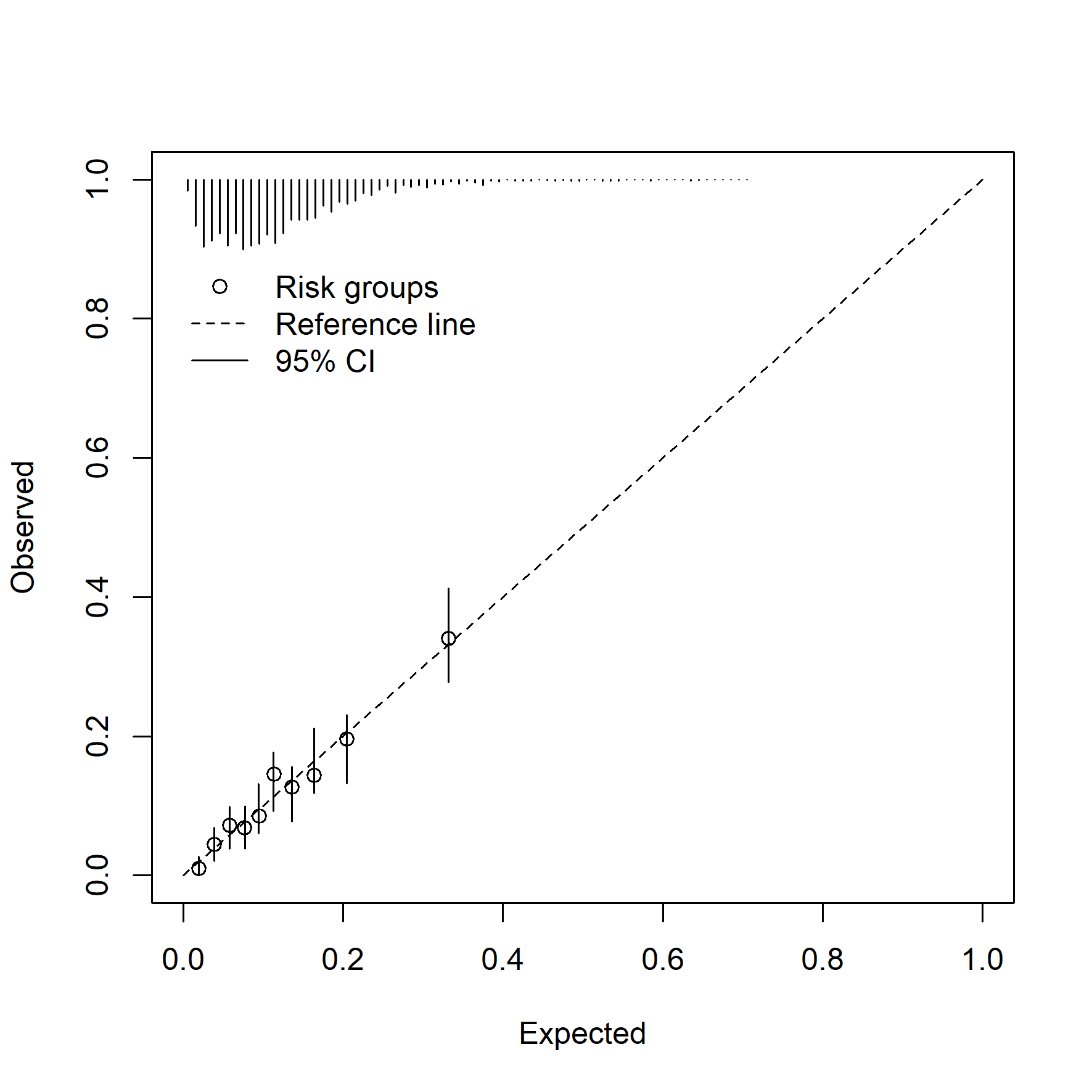


## Sensitivity Analysis: Model development using cohort data only

Supplementary Table 6: Parsimonious multivariable model for risk of transition to psychosis according to the IPD dataset (not adjusted for shrinkage) – using only the 16 cohort studies from the IPD dataset

|  | **Odds Ratio** |
| --- | --- |
| **Variable** | **(95% CI)** |
| *Intercept, log odds ratio (standard error)* | *-4.24 (0.86)* |
| Combined score: Disorganised thought content (linear) | 1.37 (1.21, 1.55) |
| Combined score: Disorganised speech (linear) | 1.32 (1.19, 1.46) |
| Global Functioning Scale (linear) | 0.97 (0.96, 0.99) |
| Genetic Risk |  |
| No | 1.00 |
| Yes | 1.58 (0.99, 2.50) |
| Treatment |  |
| No | 1.00 |
| Yes | 1.72 (0.55, 5.34) |
| Not known | 1.51 (0.46, 4.98) |

- C statistic: 0.72 (0.69, 0.74)
- E/0: 1

Supplementary Figure 4: Calibration plot comparing observed risk of transition to psychosis with that observed in the IPD dataset – cohort studies only


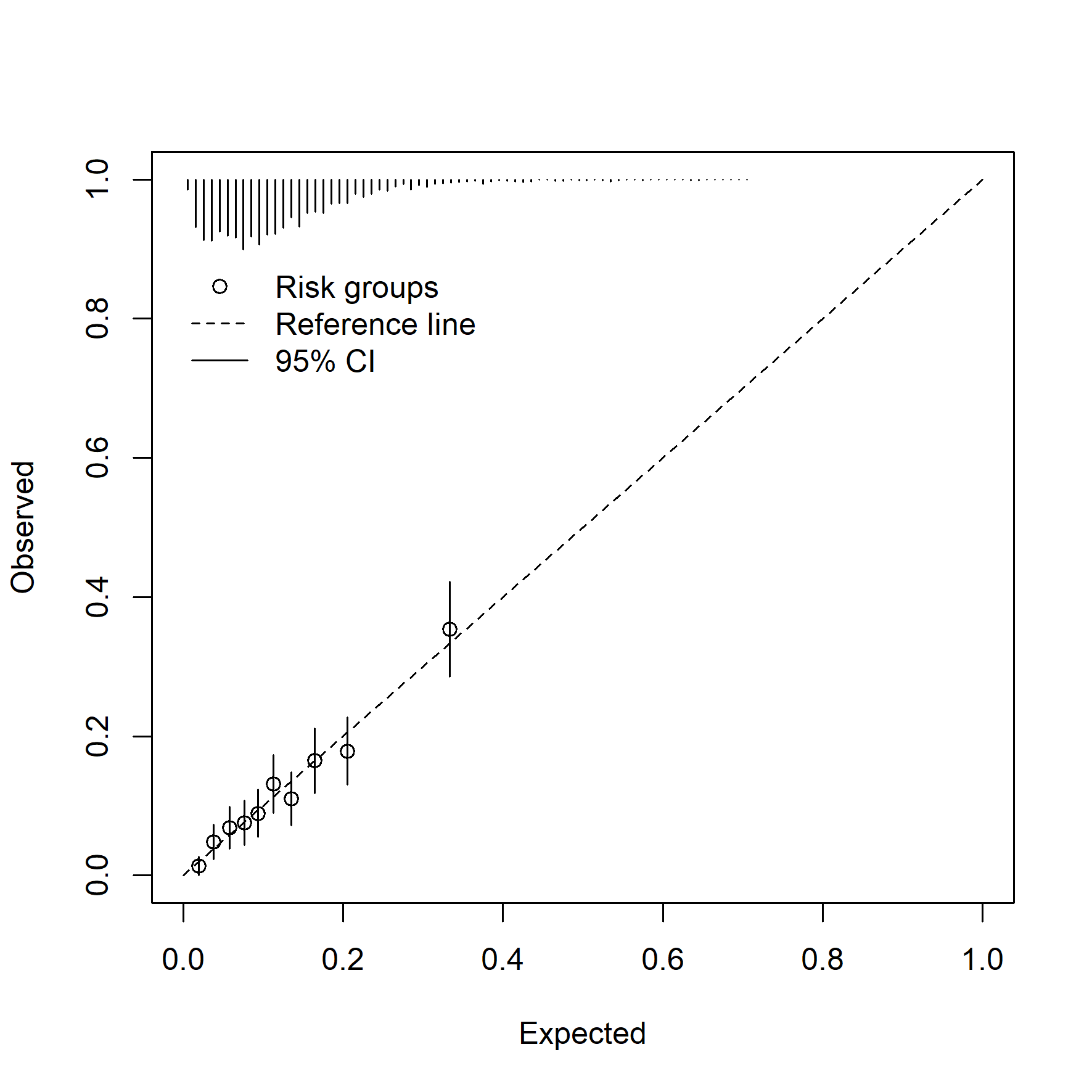


## Sensitivity Analysis: Model development using only studies with some global functioning scale data

Supplementary Table 7: Parsimonious multivariable model for risk of transition to psychosis according to the IPD dataset (not adjusted for shrinkage) – using only the 17 studies that had at least some information provided relating to global functioning

|  | **Odds Ratio** |
| --- | --- |
| **Variable** | **(95% CI)** |
| *Intercept, log odds ratio (standard error)* | *-3.58 (0.69)* |
| Combined score: Disorganised thought content (linear) | 1.37 (1.22, 1.54) |
| Combined score: Disorganised speech (linear) | 1.27 (1.17, 1.39) |
| Global Functioning Scale (linear) | 0.97 (0.96, 0.98) |
| Genetic Risk |  |
| No | 1.00 |
| Yes | 1.40 (0.96, 2.04) |
| Treatment |  |
| No | 1.00 |
| Yes | 0.80 (0.39, 1.62) |
| Not known | 0.95 (0.43, 2.11) |

- C statistic: 0.72 (0.69, 0.75)
- E/0: 1

Supplementary Figure 5: Calibration plot comparing observed risk of transition to psychosis with that observed in the IPD dataset – cohort studies only


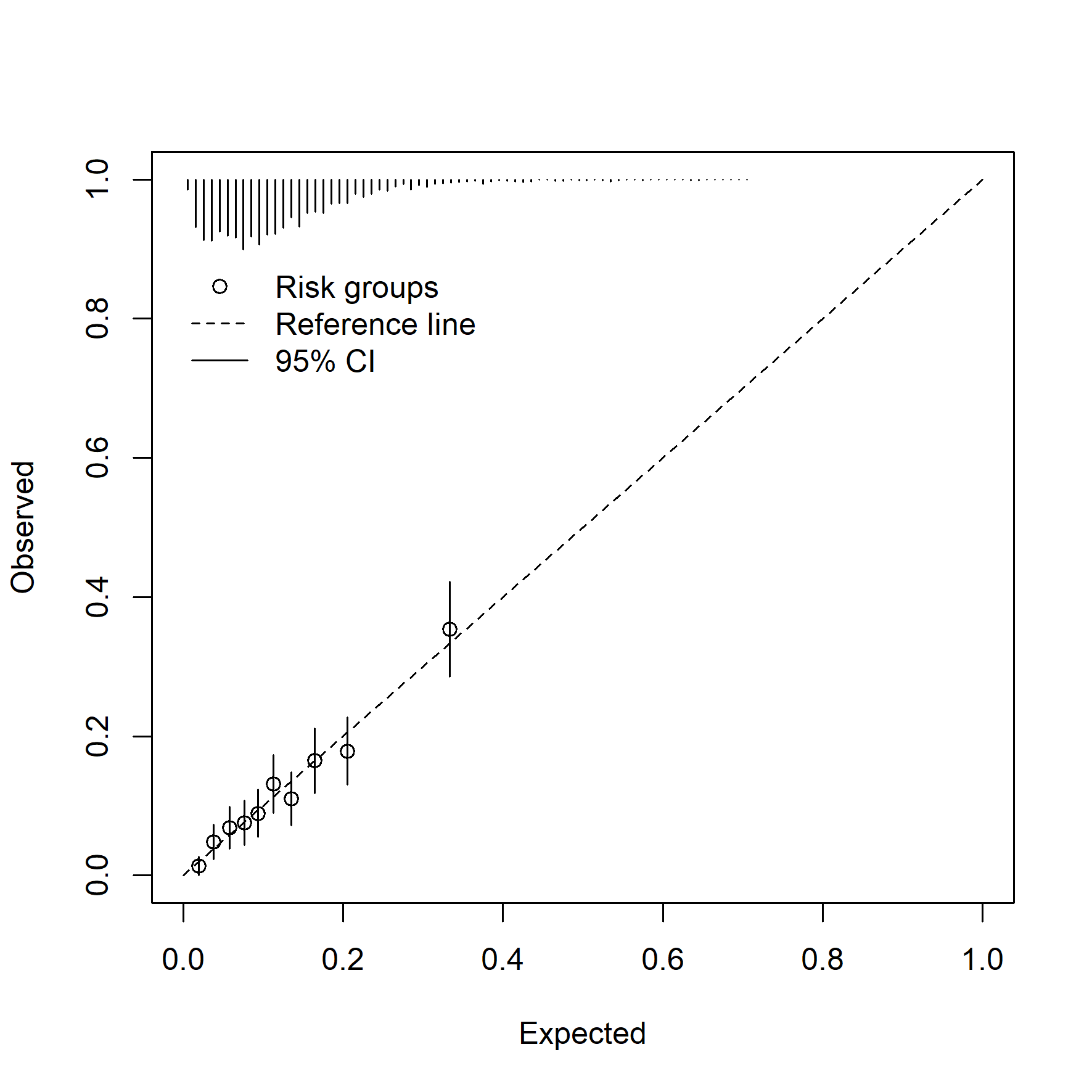

Supplement: Supplementary file 1 — Supplementary Material [file 41537_2025_582_MOESM1_ESM.docx]
